# Lenvatinib induces ferroptosis-related changes in osteosarcoma cells involving the p-STAT3/p53/xCT axis

**DOI:** 10.3389/fonc.2026.1788306

**Published:** 2026-05-29

**Authors:** Chunwang Yang, Yulong Ma, Wenxiang Shen, Xiaozhong Ma, Xiang Liu, Shaowen Du, Kaishan Ye

**Affiliations:** 1 Department of Orthopedics, The Second Hospital & Clinical Medical School, Lanzhou University, Lanzhou, Gansu, China; 2 The Second Clinical Medical School, Lanzhou University, Lanzhou, Gansu, China; 3 Key Laboratory of Orthopedics Disease of Gansu Province, The Second Hospital & Clinical Medical School, Lanzhou University, Lanzhou, Gansu, China; 4 The People’s Hospital of Linxia Hui Autonomous Prefecture, Linxia, Gansu, China; 5 Guanghe County Maternal and Child Health Hospital, Linxia, Gansu, China; 6 Gansu Provincial Hospital of Traditional Chinese Medicine, Lanzhou, Gansu, China

**Keywords:** ferroptosis, lenvatinib, osteosarcoma, p-STAT3/P53/xCT axis, targeted therapy

## Abstract

**Introduction:**

Lenvatinib is an effective antitumor agent for several solid tumors and has been used in pediatric and adolescent patients with refractory or recurrent osteosarcoma. However, the mechanism by which lenvatinib suppresses osteosarcoma progression remains incompletely defined. This study investigated whether lenvatinib induces ferroptosis-related changes in osteosarcoma cells and explored its effects on malignant biological behavior and the underlying molecular mechanism.

**Methods:**

Human osteoblast hFOB1.19 cells and osteosarcoma MG63, 143B, and U2OS cells were treated with lenvatinib. Cell viability and IC50 values were determined using CCK-8 assays. Ferroptosis-related molecular changes in 143B and U2OS cells were evaluated by qRT-PCR, Western blotting, flow cytometry, and biochemical assays measuring intracellular Fe^2+^, reduced glutathione, reactive oxygen species, and lipid peroxidation. Mitochondrial ultrastructure was examined by transmission electron microscopy, and p53 and xCT expression patterns were assessed by immunofluorescence staining. Ferroptosis inhibition assays with ferrostatin-1 were performed, and stable xCT-overexpressing osteosarcoma cell lines were generated by lentiviral transduction.

**Results:**

Lenvatinib inhibited the proliferation of 143B and U2OS cells in a concentration-dependent manner, whereas its inhibitory effect was weaker in MG63 cells and limited in hFOB1.19 osteoblasts. Transmission electron microscopy revealed ferroptosis-associated mitochondrial alterations, including mitochondrial shrinkage, loss or reduction of cristae, and increased membrane density. Lenvatinib increased intracellular Fe^2+^, reactive oxygen species, and lipid peroxidation levels while decreasing reduced glutathione, and these effects were reversed by ferrostatin-1. qRT-PCR, Western blotting, and immunofluorescence analyses showed that lenvatinib treatment was associated with increased p53 expression, reduced xCT expression, and modulation of ferroptosis-related genes and proteins. Western blotting further indicated that lenvatinib inhibited STAT3 phosphorylation, whereas ferrostatin-1 attenuated lenvatinib-induced changes in p-STAT3 and p53. Functionally, xCT upregulation enhanced osteosarcoma cell proliferation, migration, and invasion and attenuated lenvatinib-induced ferroptosis-related changes.

**Discussion:**

These findings suggest that lenvatinib suppresses osteosarcoma cell proliferation, migration, and invasion, at least in part, by inducing ferroptosis-related changes associated with modulation of the p-STAT3/p53/xCT axis. This study reveals a previously underrecognized mechanism of lenvatinib action and supports further investigation of ferroptosis-targeted strategies in osteosarcoma.

## 1 Introduction

Osteosarcoma (OS) is a primary malignant bone tumor of mesenchymal origin that predominantly affects adolescents and young adults (1). It most commonly arises in the distal femur, proximal tibia, and proximal humerus, and is characterized by early aggressive invasion and a high propensity for hematogenous dissemination, particularly to the lungs. Once pulmonary metastasis occurs, the 5-year survival rate of patients with osteosarcoma declines sharply (2, 3). At present, surgery combined with neoadjuvant chemotherapy, immunotherapy, and targeted therapy has improved postoperative survival in patients with osteosarcoma. However, these therapeutic strategies remain constrained by several unresolved limitations, including chemoresistance, poor tolerance to chemotherapy, severe treatment-related adverse reactions, local recurrence, secondary metastasis, and uncertainty regarding effective therapeutic targets and clinical response (4, 5). Thus, although conventional treatment has achieved measurable progress, its overall efficacy remains unsatisfactory because of these inherent limitations. Identifying new therapeutic strategies is therefore an urgent priority for improving prognosis and increasing long-term survival in patients with osteosarcoma.

Iron is an essential trace element required for fundamental biological processes and is indispensable for cellular metabolism and organismal homeostasis. Ferroptosis is a recently recognized form of regulated cell death that depends on iron metabolism and is driven by excessive accumulation of reactive oxygen species (ROS), lipid peroxidation, and elevated intracellular ferrous iron levels. Morphologically, ferroptotic cells typically exhibit distinctive mitochondrial alterations, including mitochondrial shrinkage, reduction or disappearance of mitochondrial cristae, and increased membrane density (6). Glutathione (GSH), composed of cysteine, glutamate, and glycine, exists mainly in reduced and oxidized forms and serves as a major antioxidant in mammalian cells. Importantly, GSH is an essential cofactor required for the activity of glutathione peroxidase 4 (GPX4). The major regulatory pathways of ferroptosis include the canonical GPX4-dependent pathway, namely the cysteine/cystine-GSH-GPX4 axis, and GPX4-independent defense systems, including the NADPH/FSP1/CoQ10 axis and the mitochondrial DHODH-mediated pathway. Both GPX4 and dihydroorotate dehydrogenase (DHODH) can regulate ferroptosis within mitochondria, whereas GPX4 also functions in the cytoplasm. DHODH can compensate for mitochondrial ferroptosis defense mediated by GPX4, while ferroptosis suppressor protein 1 (FSP1) mainly exerts its anti-ferroptotic function in the cytoplasm (7–9).

Among these mechanisms, the GPX4-dependent pathway represents the classical regulatory axis of ferroptosis and involves two principal routes: intrinsic regulation through direct inhibition of GPX4 and extrinsic regulation through suppression of system Xc^-^. Polyunsaturated fatty acids (PUFAs), which are essential substrates for lipid peroxidation, contribute to the formation of phospholipid hydroperoxides (PLOOH). GPX4, which contains a selenocysteine residue, cooperates with GSH to reduce PLOOH to phospholipid alcohols (PLOH) and hydrogen peroxide to water, thereby preventing PUFA peroxidation and ultimately suppressing ferroptotic cell death (10). System Xc^-^ is a heterodimeric cystine/glutamate antiporter that imports cystine and exports glutamate, thereby supporting intracellular GSH synthesis. It consists of the heavy-chain subunit SLC3A2/CD98hc and the light-chain subunit xCT/SLC7A11. Inhibition of system Xc^-^ by erastin or sulfasalazine suppresses GSH synthesis, inactivates GPX4, and consequently triggers ferroptosis (11–13). The tumor suppressor p53 is an upstream regulator of xCT, and modulation of xCT expression has been shown to induce ferroptosis (14). Signal transducer and activator of transcription 3 (STAT3) is closely associated with tumor initiation and progression, and inhibition of STAT3 activity can effectively restrain tumor development. Moreover, phosphorylated STAT3 (p-STAT3) can interact with p53 and suppress p53 activity (15, 16).

Accumulating evidence has established a close relationship between ferroptosis and osteosarcoma. Artemisinin exhibits anticancer activity in osteosarcoma and various other tumors by inducing ferroptosis through GPX4 inhibition and disruption of intracellular iron metabolism (17). Zoledronic acid, commonly used in patients with metastatic osteosarcoma, has been shown to promote ferroptosis in osteosarcoma cells by upregulating heme oxygenase-1 (HMOX1) and cytochrome P450 oxidoreductase; notably, HMOX1 upregulation significantly increases the expression of the ferroptosis marker PTGS2 (18, 19). In addition, tirapazamine has been reported to induce ferroptosis in osteosarcoma cells under hypoxic conditions by suppressing xCT expression, thereby exerting marked antitumor effects (20). Recent studies have further shown that lenvatinib can induce ferroptosis in hepatocellular carcinoma cells by inhibiting fibroblast growth factor receptor 4 (FGFR4), and can promote ferroptosis in endothelial cells, suppress YAP expression, and contribute to elevated blood pressure (21, 22).

Despite these advances, a critical question remains unresolved: whether lenvatinib can induce ferroptosis in osteosarcoma cells, and if so, through which molecular mechanism. Although ferroptosis has been increasingly implicated in osteosarcoma biology, the role of lenvatinib as a ferroptosis inducer in osteosarcoma has not been clearly defined. In particular, whether lenvatinib regulates xCT-mediated ferroptosis and thereby alters the malignant biological behavior of osteosarcoma cells remains unknown. Based on this gap, the present study was designed to investigate the effects of lenvatinib on osteosarcoma cells and to evaluate whether ferroptosis-related changes are involved in lenvatinib-mediated suppression of osteosarcoma cell proliferation, migration, and invasion. Therefore, this study aimed to investigate whether lenvatinib induces ferroptosis-related changes in osteosarcoma cells and to explore whether these effects are associated with xCT regulation and modulation of the p-STAT3/p53/xCT axis.

## 2 Material and methods

### 2.1 Reagents and cells

Lenvatinib was purchased from MCE (USA), encapsulated, and stored to prevent repeated freeze-thaw cycles. Ferrostatin-1 (Fer-1) was acquired from Sigma (USA) and prepared fresh prior to use. The β-actin mouse antibody (TA-09, 1:1000), goat anti-mouse secondary antibody (ZB-2305, 1:10000), and goat anti-rabbit secondary antibody (ZB-2301, 1:10000) were sourced from Beijing Zhongshan Jinqiao Biotechnology Co., Ltd. The xCT rabbit antibody (T57046, 1:1000), GPX4 rabbit antibody (T56959, 1:1000), and p-STAT3 rabbit antibody (T56566, 1:1000) were obtained from Shanghai Abmart Pharmaceutical Technology Co., Ltd. p53 mouse antibody (60283-2-IG, 1:5000) and STAT3 rabbit antibody (10253-2-AP, 1:5000) were purchased from Wuhan Sanying Biotechnology Co., Ltd. Fluorescent goat anti-mouse secondary antibody (BD9276, 1:200) and fluorescent goat anti-rabbit secondary antibody (BD9279, 1:400) were acquired from Suzhou Boaolong Technology Co., Ltd. The human osteoblast cell line hFOB1.19 and human osteosarcoma cell lines MG63, 143B, and U2OS were obtained from Punuosai Life Science Technology Co., Ltd. Cells were cultured in 10% DMEM complete medium at 37 °C with 5% CO_2_ in a cell incubator.

### 2.2 Cell viability

Cell viability was assessed using the CCK-8 Cell Counting Kit (BaiSha, China). Under light-protected conditions, 10 μl of CCK-8 solution and 90 μl of serum-free culture medium were added to each well with the pre-mixed solution. Careful attention was paid to ensure that no air bubbles were present, as they could interfere with the experimental results. The absorbance (OD) values of each well were measured at 450 nm using a microplate reader (Bio Tek, USA). Subsequently, the cell viability and the half-maximal inhibitory concentration (IC50) of the drug were calculated based on the obtained data. Cells were seeded into 96-well plates at a density of 5 × 10³ cells/well and allowed to adhere overnight. After treatment with lenvatinib for 24, 48, or 72 h, 10 μL CCK-8 reagent was added to each well and incubated for 2h at 37 °C protected from light. Absorbance was measured at 450 nm using a microplate reader (Bio-Tek, USA). Cell viability was normalized to the control group. IC50 values were calculated in GraphPad Prism 10.1.2 by nonlinear regression using a four-parameter logistic model (log[inhibitor] vs. normalized response, variable slope).

### 2.3 TEM

Initially, cell samples were processed by adding 2.5% glutaraldehyde fixative to resuspend the cells. Following this, the cells were washed with 0.1M phosphate-buffered solution and encapsulated in agarose. Subsequently, the samples were fixed in 1% osmium tetroxide under light-protected conditions at room temperature for 2 hours. A series of dehydration steps was performed using increasing concentrations of ethanol (30%-100%). The samples were then infiltrated with a 1:1 mixture of acetone and embedding medium. Ultra-thin sections, ranging from 60 to 80 nm in thickness, were cut using an ultramicrotome. The sections were stained with a 2% uranyl acetate alcohol solution under light protection, followed by staining with 2.6% lead citrate in the absence of carbon dioxide. Finally, the samples were observed and photographed using a transmission electron microscope (Hitachi, Japan).

### 2.4 RNA extraction, reverse transcription and RT-qPCR

Total RNA was extracted according to the instructions provided with the Trizol reagent (AG RNAex Pro RNA extraction kit). Subsequently, cDNA was synthesized by reverse transcription of the total RNA using the Evo M-MLV Reverse Transcription Kit (AikeRui, China), which includes gDNA Clean Reagent, Evo M-MLV RTase Enzyme Mix, and other reagents. GAPDH was used as the internal control, and amplification was carried out using primers (Qingke, China) and the SYBR Green Pro Taq HS Premix qPCR Kit (AikeRui, China). Gene expression analysis was performed based on the amplification results, and the primer sequences used are listed in **Table 1**.

**Table 1:** Reverse transcription quantitative polymerase chain reaction primer sequences.

GeneID	Forward primer sequences (5′-3′)	Reverse primer sequences (5′-3′)
GAPDH	ACCCACTCCTCCACCTTTGAC	TCCACCACCCTGTTGCTGTAG
p53	CCTCCTCAGCATCTTATCCGAGTG	CCAACCTCAGGCGGCTCATAG
PTGS2	CCATTGACCAGAGCAGGCAGATG	TCTACCAGAAGGGCAGGATACAGC
xCT	TTTGTTGCCCTCTCCTGCTTTG	AGTGTGCTTGCGGACATGAATC
GPX4	CCGCTGTGGAAGTGGATGAAG	TGTCGATGAGGAACTGTGGAGAG

### 2.5 Western blotting

Cells with good morphology were treated with lenvatinib at the indicated concentrations for 24 h. All protein extraction procedures were performed on ice. Cells were washed twice with PBS and lysed with 500 μL ice-cold lysis buffer consisting of RIPA: PMSF:phosphatase inhibitor cocktail = 100:1:2 (Beyotime, China). After incubation on ice for 10 min, cells were scraped and briefly sonicated at low frequency. Lysates were centrifuged at 12,000 rpm for 20 min at 4 °C, and the supernatants were collected. Protein concentrations were determined using a BCA assay (BCA reagents A:B = 50:1, incubated at 37 °C for 30 min, absorbance read at 562 nm) using BSA standards to generate a standard curve.

Protein samples were mixed with 5×loading buffer 4:1, v/v and denatured at 100 °C for 10 min. Equal amounts of protein were separated by 10% SDS-PAGE and transferred onto PVDF membranes (Merck, USA), which were activated in methanol for 1 min prior to use. Protein transfer was performed in ice-cold transfer buffer at a constant current of 350 mA, with transfer time adjusted according to the molecular weight of the target protein. Membranes were blocked with Quick Blocking Buffer (Beyotime, China) for ~40 min at low temperature with gentle shaking (65 rpm), followed by washing with TBST (3 × 10 min, 100 rpm). Membranes were then incubated with primary antibodies overnight (8 h) at low temperature (65 rpm), washed with TBST (3 × 10 min), and incubated with HRP-conjugated secondary antibodies for 1 h (65 rpm). After additional TBST washes (3 × 10 min), bands were visualized using an enhanced chemiluminescence (ECL) kit (BaiSha, China) and an automatic imaging system. Band intensities were quantified using ImageJ, and β-actin was used as the loading control.

### 2.6 Immunofluorescence

Cells were seeded onto sterile round glass coverslips for cell adhesion. Subsequently, 1 mL of 4% paraformaldehyde solution (BaiSha, China) was added for fixation, followed by permeabilization with 0.2% Triton X-100 (solarbio, China) at room temperature. The samples were then blocked with 5% goat serum (Solarbio, China) at room temperature. Afterward, 200 μL of primary antibody solution was added per well, and the samples were incubated overnight at 4 °C. Following this, 200 μL of secondary antibody solution was added per well, and the samples were incubated at room temperature for 1 hour in the dark. The coverslips were then removed, and 10-20 μL of DAPI-containing antifade reagent (solarbio, China) was applied. The coverslips were carefully placed on glass slides, and the samples were observed and photographed using a fluorescence upright microscope (Olympus, Japan).

### 2.7 ROS assay

The ROS detection was conducted using a ROS detection kit (Beyotime, China). First, the DCFH-DA probe was diluted to a final concentration of 10 μM. After adding 2 mL per well of the diluted probe to the cells, the cells were incubated in the dark at 37 °C and 5% CO2 for 30 minutes. Following the incubation, the cells were washed twice with serum-free culture medium to remove any unincorporated DCFH-DA probe. After digestion with trypsin (without EDTA) and resuspension in serum-free medium, the fluorescence intensity was measured using a flow cytometer (Beckman, USA) with an excitation wavelength of 488 nm and an emission wavelength of 525 nm.

### 2.8 Lipid oxidation detection

Lipid oxidation detection was conducted using a Lipid Peroxidation MDA Assay Kit (Beyotime, China). The required TBA storage solution, MDA detection working solution, and standard samples of varying concentrations were prepared. On ice, 150 μL of Western and IP cell lysis buffer was added per well to effectively lyse the cells. For each 100 μL of sample to be measured, along with different concentration standards and lysis buffer blank controls, 200 μL of MDA detection working solution was added. The tubes were sealed with adhesive film and tightly weighted down. The samples were then heated in a 100 °C metal bath under dark conditions. After the samples were cooled to room temperature in a water bath, they were centrifuged at 1000 rpm for 10 minutes. The supernatant was carefully collected, and 200 μL of the supernatant was transferred into a 96-well plate. The absorbance was measured at 532 nm using a microplate reader.

### 2.9 Ferrous iron assay

The cell ferrous colorimetric assay kit (Elabscience, China) was used for testing. Prior to the assay, the reagents in the kit were equilibrated to room temperature. Standard iron solutions of 100 μmol/L were prepared, and different concentrations of the standards were diluted as required. Cells were collected according to the cell passage procedure. Approximately 1 × 10^6 cells were added to 0.2 mL of Reagent 1, mixed thoroughly, and then placed on ice for lysis. After centrifugation, the supernatant was collected for measurement. For the standard wells, 80 μL of the different concentrations of standard solutions was mixed with 80 μL of Reagent 3 (color development solution). For the test wells, 80 μL of the experimental sample was added along with 80 μL of Reagent 3. For the control wells, 80 μL of the control sample was mixed with 80 μL of Reagent 2 (control solution). The mixture was well mixed and incubated at 37 °C. Finally, the absorbance was measured at 593 nm using a microplate reader.

### 2.10 GSH assay

The microquantitative reduced GSH assay kit (Nanjing Jiancheng, China) was used for the experiment. The required GSH standard solvent solution, 1 mmol/L GSH standard solution, and 20 μmol/L GSH standard solution were prepared. Cells were collected according to the cell passage procedure and cell pellets were obtained. The cells were resuspended in 0.4 mL PBS, and ultrasound was used for thorough cell disruption. A 0.1 mL aliquot of the disrupted cell suspension was mixed with 0.1 mL of Reagent 1 (precipitating agent). After centrifugation, the supernatant was collected for measurement. For the blank wells, 100 μL of Reagent 1, 100 μL of Reagent 2 (buffer), and 25 μL of Reagent 3 (color development reagent) were added. For the standard wells, 100 μL of 20 μmol/L GSH standard solution, 100 μL of Reagent 2, and 25 μL of Reagent 3 were added. For the test wells, 100 μL of the supernatant, 100 μL of Reagent 2, and 25 μL of Reagent 3 were added. The mixtures were thoroughly mixed and left to stand at room temperature. Finally, the absorbance was measured at 405 nm using a microplate reader.

### 2.11 Construction of up-regulated xCT osteosarcoma cells by stable transfection

Cells were seeded into six-well plates at a density of 1 × 10^4 cells per well. Once the cell density reached approximately 40% confluence, they were transfected with oe-xCT and oe-NC lentivirus (JiMa, China). After 8 to 12 hours of incubation, cells were observed for morphological changes. If any changes in morphology were detected, the medium was replaced. If no significant differences in cell status were observed compared to the uninfected control group, indicating that the lentivirus did not exhibit apparent cytotoxicity, the medium was not replaced and cells were cultured further. After 24 hours, fresh culture medium was added. Due to the rapid growth of 143B cells, a complete culture medium without penicillin-streptomycin (5% solution) was used. Fluorescent expression was monitored 72 to 96 hours post-infection. When the lentivirus-transfected cells reached approximately 85% confluence, the culture medium was replaced with a complete medium containing 5 μg/mL puromycin. Every 24 hours, fluorescence was observed using a fluorescence inverted microscope, and the medium was replaced with puromycin-containing complete medium until stable lentivirus-transfected clones were successfully selected. Finally, the efficiency of xCT upregulation was quantitatively validated by RT-qPCR and western blotting. For RT-qPCR, the mRNA levels of SLC7A11 (xCT) were normalized to GAPDH and calculated using the 2^−ΔΔCt method. For western blotting, xCT protein levels were normalized to β-actin, and band intensities were quantified using ImageJ. Fluorescence was monitored during the selection period to evaluate transduction efficiency when applicable.

### 2.12 Wound healing assay

Initially, a 200 μL pipette tip was used to create a scratch in the six-well plate, with the tip held vertically against the plate and the back surface to make the scratch. After the scratching procedure, the cells were washed twice with PBS. The scratch area was then photographed using a standard inverted microscope (Olympus, Japan) to record the initial (0-hour) scratch area. Subsequently, the cells were cultured at 37 °C with 5% CO2 for 24 hours, after which the scratch area was photographed again. The relative migration area was calculated using the following formula: relative migration area = (0-hour scratch area - 24-hour scratch area)/0-hour scratch area. After scratching, cells were washed and cultured in serum-free medium during the wound-healing period to minimize proliferation-related closure.

### 2.13 Transwell migration and invasion test

#### 2.13.1 Migration assay

8.0 μm pore size Transwell chambers (Corning, USA) were placed into 24-well plates. Complete culture medium (800 μL) was added to the lower compartment of each Transwell. After the intervention, cells were passaged and collected by centrifugation. The cells were resuspended in serum-free medium, counted, and adjusted to a density of 1 × 10^5 cells/mL. A volume of 200 μL of the prepared cell suspension was added to the upper compartment of the Transwell chamber. The chambers were then incubated at 37 °C with 5% CO2 for 24 hours. After the incubation, the Transwell chambers were removed, the old medium discarded, and the chambers gently washed with PBS. Following this, 500 μL of 4% paraformaldehyde was added to fix the cells in the chamber. The fixation solution was then discarded, and the chambers were washed with PBS. The cells were subsequently stained using 0.1% crystal violet solution. After staining, the chambers were washed with PBS, and the upper compartment was gently wiped with a cotton swab. After air drying, the cells were observed and photographed using an inverted microscope at 200× magnification.

#### 2.13.2 Invasion assay

Matrigel (BD Biosciences, USA), pre-cooled at 4 °C, was mixed with serum-free culture medium at a ratio of 1:6. A volume of 60 μL of this mixture was added to the upper compartment of the Transwell chamber. The chamber was then incubated in a 37 °C incubator to allow for gelation. After incubation, the excess liquid was discarded. The upper compartment was then filled with 100 μL of serum-free medium and placed back into the incubator for further hydration of the basement membrane. The remaining steps were performed as described in the Transwell migration assay.

### 2.14 Statistical analysis

Experimental data were analyzed using GraphPad Prism 10.1.2, and image analysis was performed with Image J software. Dose–response curves were fitted by nonlinear regression (four-parameter logistic model with variable slope), and IC50 values were derived from the fitted curves. The statistical results are presented as the mean ± standard deviation (X ± SD) of three independent experiments. Comparisons between two groups were performed using Student’s t-test, whereas comparisons among multiple groups were performed using one-way ANOVA. A p-value of less than 0.05 was considered statistically significant.

## 3 Results

### 3.1 Lenvatinib suppressed the proliferation of osteosarcoma cells

To evaluate the effect of lenvatinib on cell proliferation, human osteosarcoma U2OS, 143B, and MG63 cells, as well as human osteoblast hFOB1.19 cells, were treated with different concentrations of lenvatinib for 24, 48, and 72 h. Cell proliferative activity was then assessed using the CCK-8 assay.

**Figure 1:**
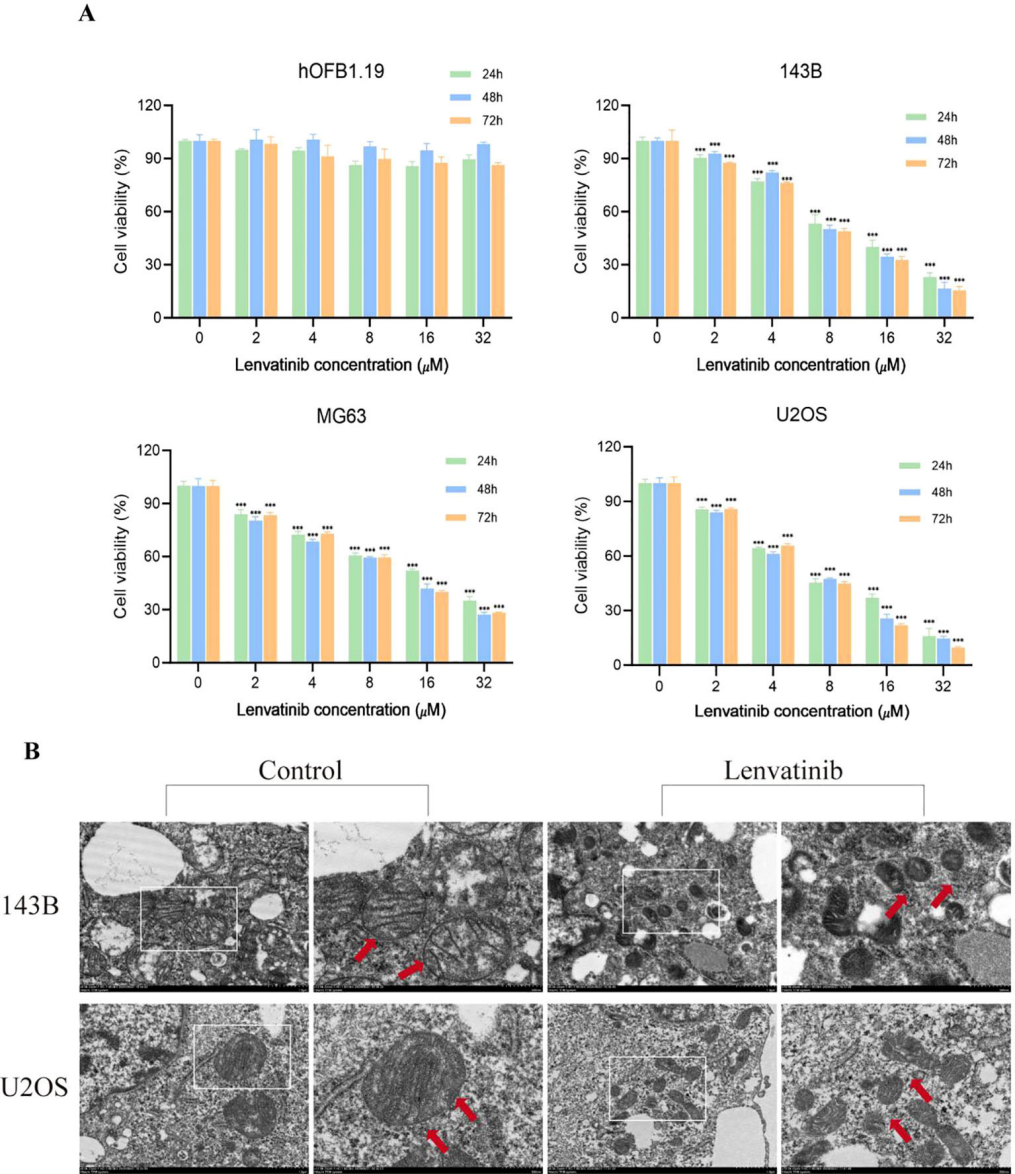
Lenvatinib inhibited osteosarcoma cell proliferation in a concentration-dependent manner. **(A)** Cell viability of hFOB1.19, 143B, MG63, and U2OS cells was assessed using the CCK-8 assay after treatment with lenvatinib for 24, 48, and 72 h. Data are presented as the mean ± standard deviation (SD) from three independent experiments (n = 3). *p < 0.05, **p < 0.01, ***p < 0.001. **(B)** TEM analysis of the effects of lenvatinib on mitochondrial morphology in osteosarcoma cells. U2OS and 143B cells were treated with lenvatinib at concentrations of 5 μM and 7 μM, respectively, for 24 h. Mitochondrial ultrastructural changes were observed by transmission electron microscopy (TEM).

As shown in **Figure 1A**, after treatment with lenvatinib for 24, 48, and 72 h, the IC50 values were 7.049, 7.385, and 7.918 μM in 143B cells, respectively; 11.82, 14.64, and 10.83 μM in MG63 cells, respectively; and 4.956, 5.103, and 5.756 in U2OS cells, respectively. These results showed that lenvatinib inhibited the proliferation of all three osteosarcoma cell lines in a concentration-dependent manner. The inhibitory effect was more pronounced in 143B and U2OS cells than in MG63 cells, whereas lenvatinib had only a limited effect on the proliferation of human osteoblast hFOB1.19 cells.

In 143B and U2OS cells, the inhibitory effect of lenvatinib after 48 or 72 h of treatment was not stronger than that observed after 24 h of treatment. Therefore, 143B and U2OS cells treated with lenvatinib for 24 h were selected for subsequent experiments. These findings indicate that lenvatinib exerted a concentration-dependent inhibitory effect on osteosarcoma cell proliferation, while showing limited cytotoxicity toward hFOB1.19 osteoblasts under the tested *in vitro* conditions.

A. Cell viability of hFOB1.19, 143B, MG63, and U2OS cells was assessed using the CCK-8 assay after treatment with lenvatinib for 24, 48, and 72 h. Data are presented as the mean ± standard deviation (SD) from three independent experiments (n = 3). *p < 0.05, **p < 0.01, ***p < 0.001.

B. TEM analysis of the effects of lenvatinib on mitochondrial morphology in osteosarcoma cells. U2OS and 143B cells were treated with lenvatinib at concentrations of 5 μM and 7 μM, respectively, for 24 h. Mitochondrial ultrastructural changes were observed by transmission electron microscopy (TEM).

### 3.2 Lenvatinib induced mitochondrial morphological alterations in osteosarcoma cells

To determine whether lenvatinib treatment altered the ultrastructure of osteosarcoma cells, 143B and U2OS cells were examined by transmission electron microscopy (TEM). TEM analysis showed that lenvatinib-treated osteosarcoma cells exhibited characteristic mitochondrial morphological changes associated with ferroptosis, including mitochondrial shrinkage, reduced mitochondrial volume, increased membrane density compared with the control group, and a decrease or even loss of mitochondrial cristae (**Figure 1B**).

### 3.3 Lenvatinib regulated ferroptosis-related phenotypic changes in osteosarcoma cells

#### 3.3.1 Lenvatinib induced a concentration-dependent increase in ROS levels in osteosarcoma cells

ROS accumulation is an important contributor to ferroptosis. To determine whether lenvatinib affected intracellular ROS levels in osteosarcoma cells, 143B and U2OS cells were treated with different concentrations of lenvatinib, and ROS levels were measured by flow cytometry using the DCFH-DA fluorescent probe.

**Figure 2:**
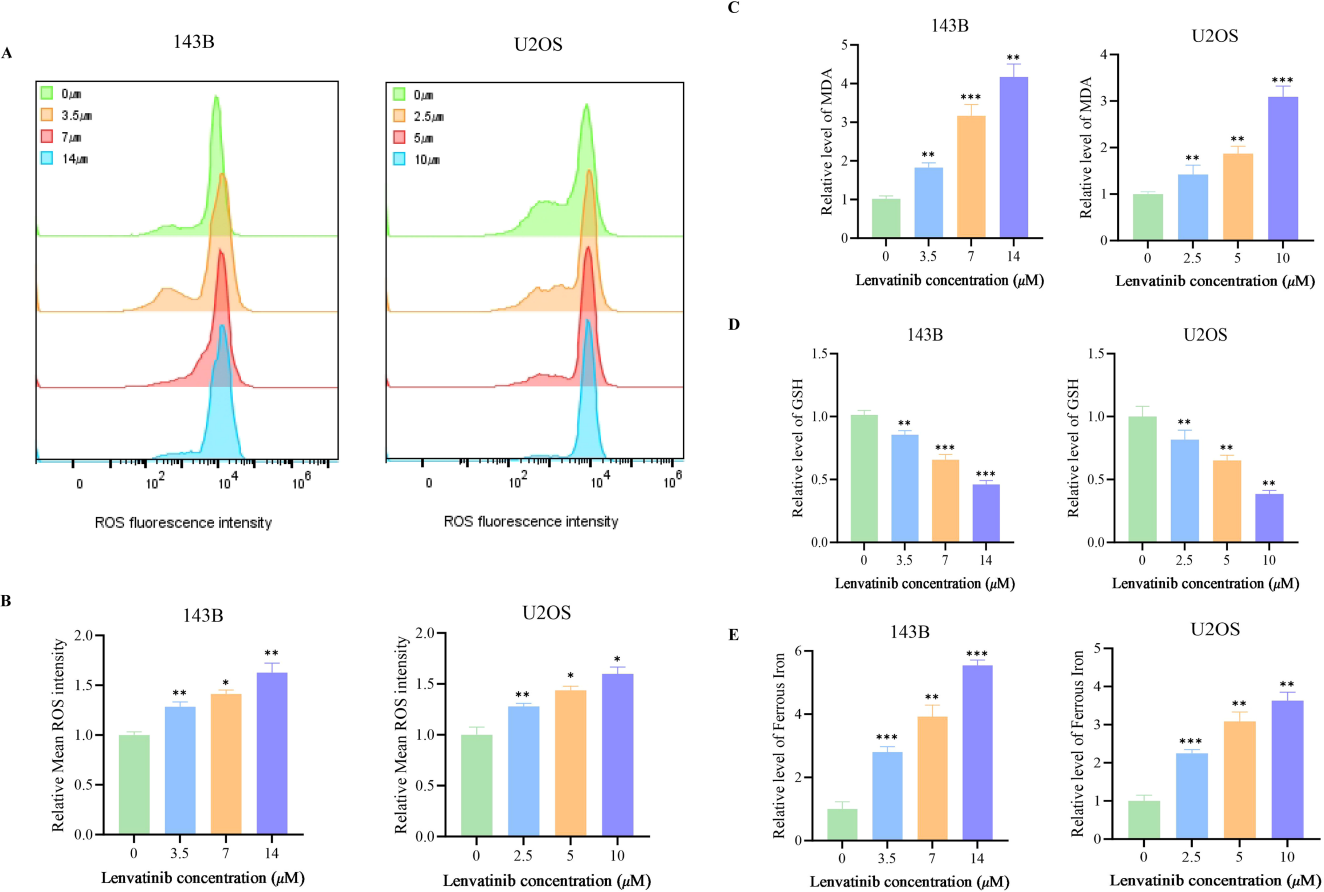
Lenvatinib increased ROS levels in osteosarcoma cells. Cells were treated with lenvatinib for 24 h. The treatment concentrations were 0, 2.5, 5, and 10 μM for U2OS cells, and 0, 3.5, 7, and 14 μM for 143B cells. **(A)** Flow cytometry analysis of ROS levels in 143B and U2OS cells. **(B)** The mean fluorescence intensity of ROS in 143B and U2OS cells increased in a concentration-dependent manner. **(C)** Lenvatinib regulates changes in MDA levels in osteosarcoma cells. The MDA levels in 143B and U2OS cells increased in a concentration-dependent manner. **(D)** Lenvatinib regulates changes in reduced GSH levels in osteosarcoma cells. The levels of reduced GSH in 143B and U2OS cells decreased in a concentration-dependent manner. **(E)** Lenvatinib regulates changes in ferrous ion (Fe^2+^) levels in osteosarcoma cells. The intracellular ferrous ion levels in 143B and U2OS cells increased in a concentration-dependent manner. Data are presented as the mean ± standard deviation (SD) (n = 3). *p < 0.05, **p < 0.01, ***p < 0.001.

The results showed that the mean fluorescence intensity increased progressively with increasing lenvatinib concentrations, indicating a significant elevation of intracellular ROS levels in osteosarcoma cells (**Figures 2A, B**).

#### 3.3.2 Lenvatinib increased MDA levels in osteosarcoma cells

Previous studies have shown that lipid peroxidation of polyunsaturated fatty acid-containing phospholipids, which disrupts the phospholipid bilayer of the cell membrane, is a major event in ferroptosis. Malondialdehyde (MDA) is a naturally generated product of lipid peroxidation; therefore, intracellular MDA levels can be used to evaluate the extent of lipid oxidative damage.

To determine whether lenvatinib affected lipid peroxidation in osteosarcoma cells, 143B and U2OS cells were treated with different concentrations of lenvatinib. Intracellular MDA levels were quantified using a colorimetric assay based on the reaction between MDA and thiobarbituric acid (TBA), which produces a red-colored product. The absorbance was measured using a microplate reader. The results showed that intracellular MDA levels increased markedly with increasing lenvatinib concentrations, indicating enhanced lipid peroxidation in osteosarcoma cells (**Figure 2C**).

#### 3.3.3 Lenvatinib decreased reduced GSH levels in osteosarcoma cells in a concentration-dependent manner

Reduced glutathione (GSH) is one of the major intracellular forms of glutathione and serves as an essential cofactor for GPX4 activation. To determine whether lenvatinib affected intracellular reduced GSH levels, 143B and U2OS cells were treated with different concentrations of lenvatinib.

Reduced GSH levels were quantitatively measured using a colorimetric assay based on the reaction between reduced GSH and 5,5′-dithiobis-(2-nitrobenzoic acid) (DTNB), which generates a yellow-colored product. Absorbance was measured using a microplate reader.

The results showed that intracellular reduced GSH levels decreased markedly as the lenvatinib concentration increased (**Figure 2D**).

#### 3.3.4 Lenvatinib increased intracellular ferrous iron levels in osteosarcoma cells in a concentration-dependent manner

Ferroptosis is closely associated with iron metabolism. Excessive intracellular accumulation of ferrous iron (Fe^2+^) can promote oxidative stress and contribute to ferroptotic cell death. To determine whether lenvatinib affected intracellular Fe^2+^ levels, 143B and U2OS cells were treated with different concentrations of lenvatinib.

Intracellular Fe^2+^ levels were quantitatively measured using a ferrous iron detection probe, and the signal intensity was detected using a microplate reader. The results showed that intracellular Fe^2+^ levels increased significantly with increasing lenvatinib concentrations in osteosarcoma cells (**Figure 2E**).

### 3.4 Fer-1 attenuated lenvatinib-induced ferroptosis-related changes in osteosarcoma cells

To further evaluate whether ferroptosis contributed to lenvatinib-induced cell death in osteosarcoma cells, osteosarcoma cells were co-treated with lenvatinib and the ferroptosis inhibitor ferrostatin-1 (Fer-1), and a ferroptosis inhibition assay was performed.

#### 3.4.1 The ferroptosis inhibitor Fer-1 reversed lenvatinib-induced cell death in osteosarcoma cells

To determine whether Fer-1 could reverse the reduction in cell viability induced by lenvatinib in 143B and U2OS cells, cell viability was measured using the CCK-8 assay after co-treatment with Fer-1 and lenvatinib.

**Figure 3:**
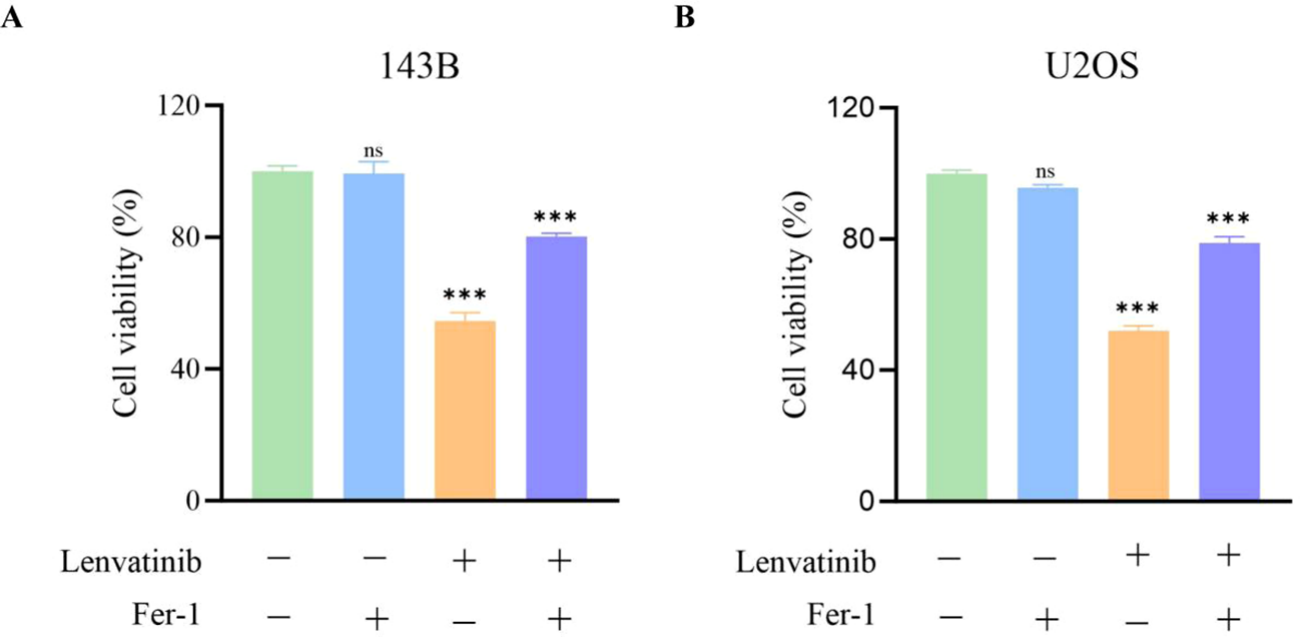
Fer-1 reversed lenvatinib-induced loss of cell viability in osteosarcoma cells. Cells were pretreated with Fer-1 for 1 h and then co-treated with lenvatinib for 24 h. The concentration of Fer-1 was 5 μM. Lenvatinib was used at 5 μM in U2OS cells and 7 μM in 143B cells. **(A)** Fer-1 reversed lenvatinib-induced cell death in 143B cells. **(B)** Fer-1 reversed lenvatinib-induced cell death in U2OS cells. Data are presented as the mean ± standard deviation (SD) (n = 3). ns, not significant; *p < 0.05, **p < 0.01, ***p < 0.001.

The results showed that, compared with the lenvatinib-treated group, cell viability was markedly restored in the Fer-1 plus lenvatinib co-treatment group. In contrast, Fer-1 alone had no obvious effect on osteosarcoma cell viability compared with the control group (**Figure 3**). These findings indicate that the ferroptosis inhibitor Fer-1 reversed lenvatinib-induced cell death in osteosarcoma cells, supporting the involvement of ferroptosis-related cell death in the response of osteosarcoma cells to lenvatinib.

#### 3.4.2 Fer-1 reversed lenvatinib-induced ferroptosis-related phenotypic changes in osteosarcoma cells

To determine whether the ferroptosis inhibitor Fer-1 could reverse the ferroptosis-related phenotypic changes induced by lenvatinib in 143B and U2OS cells, ferroptosis-related indicators were measured after co-treatment with Fer-1 and lenvatinib using flow cytometry and biochemical assays.

**Figure 4:**
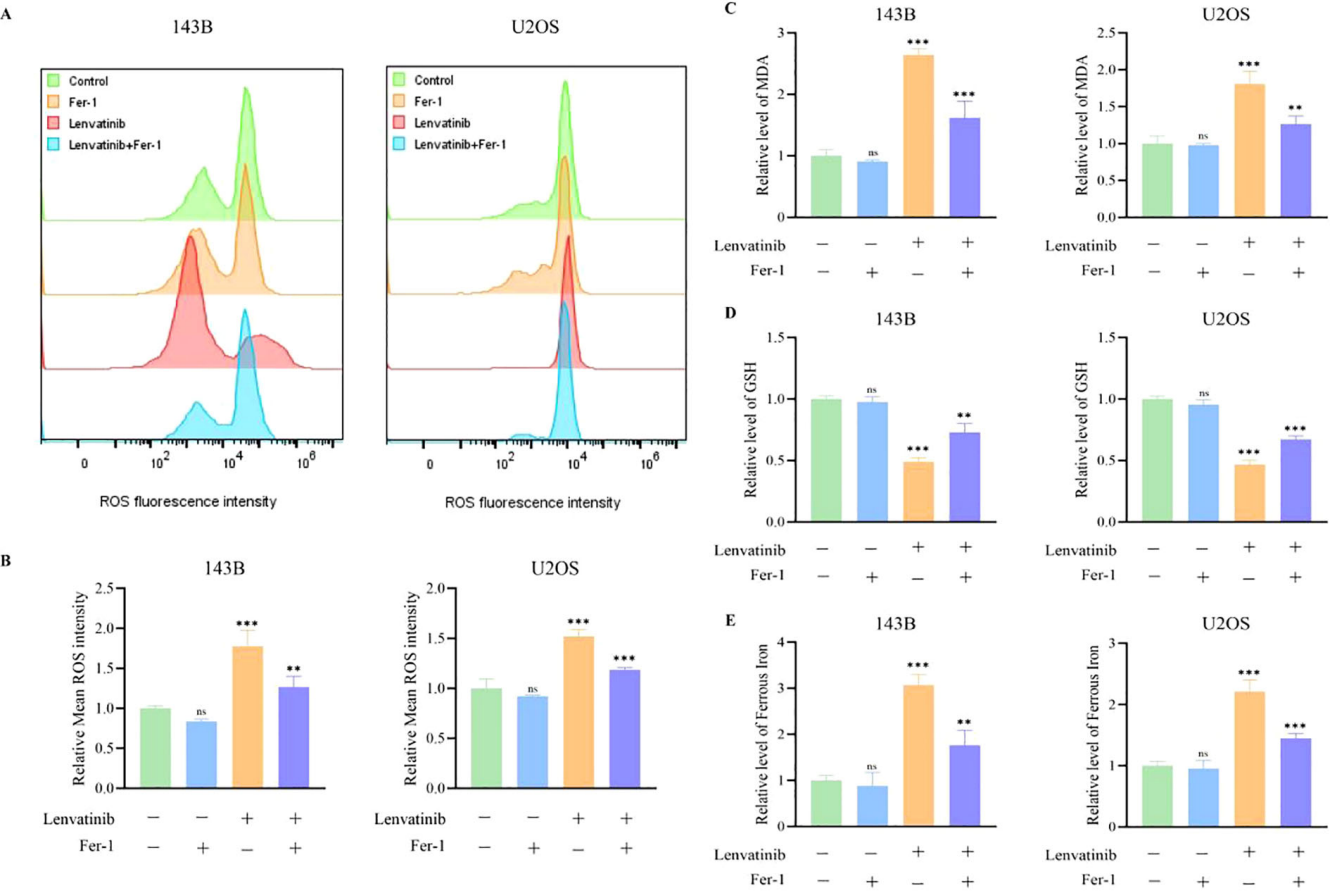
Fer-1 reversed lenvatinib-induced ferroptosis-related phenotypic changes in osteosarcoma cells. Cells were pretreated with Fer-1 for 1 h and then co-treated with lenvatinib for 24 h. The concentration of Fer-1 was 5 μM. Lenvatinib was used at 5 μM in U2OS cells and 7 μM in 143B cells. **(A)** Representative ROS fluorescence intensity and quantitative analysis of mean fluorescence intensity in 143B cells following co-treatment with Fer-1 and lenvatinib. **(B)** Representative ROS fluorescence intensity and quantitative analysis of mean fluorescence intensity in U2OS cells following co-treatment with Fer-1 and lenvatinib. **(C)** Changes in MDA levels in 143B and U2OS cells following co-treatment with Fer-1 and lenvatinib. **(D)** Changes in reduced GSH levels in 143B and U2OS cells following co-treatment with Fer-1 and lenvatinib. **(E)** Changes in intracellular ferrous iron levels in 143B and U2OS cells following co-treatment with Fer-1 and lenvatinib. Data are presented as the mean ± standard deviation (SD) (n = 3). ns, not significant; *p < 0.05, **p < 0.01, ***p < 0.001.

The results showed that, compared with the lenvatinib group, the Fer-1 plus lenvatinib co-treatment group exhibited reduced mean fluorescence intensity, indicating decreased ROS levels in osteosarcoma cells (**Figures 4A, B**). In addition, MDA and intracellular ferrous iron levels were decreased (**Figures 4C, E**), whereas reduced GSH levels were increased (**Figure 4D**). By contrast, Fer-1 alone did not significantly affect these ferroptosis-related phenotypes compared with the control group. These findings indicate that Fer-1 effectively reversed the ferroptosis-related phenotypic alterations induced by lenvatinib in osteosarcoma cells.

### 3.5 Lenvatinib-induced ferroptosis-related changes were associated with modulation of the p-STAT3/p53/xCT axis

#### 3.5.1 Lenvatinib regulated the expression of ferroptosis-related genes, which was reversed by Fer-1

To investigate the involvement of ferroptosis-related genes in lenvatinib-induced ferroptosis in 143B and U2OS cells, the expression levels of p53, GPX4, xCT, and PTGS2 were examined by qRT-PCR after lenvatinib treatment.

**Figure 5:**
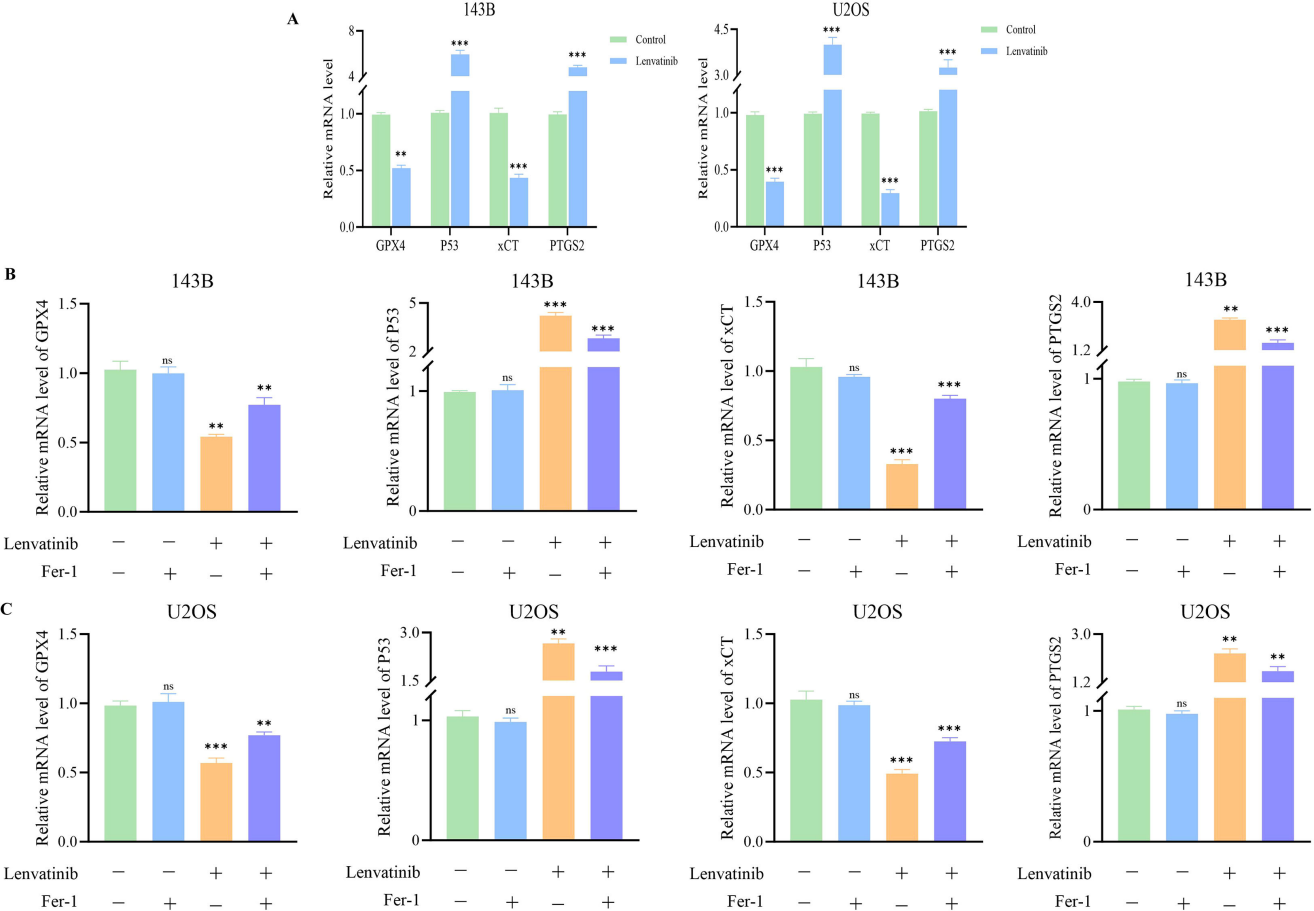
Lenvatinib regulated ferroptosis-related gene expression in osteosarcoma cells, and these effects were reversed by Fer-1. Cells were pretreated with Fer-1 for 1 h and then co-treated with lenvatinib for 24 h. The concentration of Fer-1 was 5 μM. Lenvatinib was used at 5 μM in U2OS cells and 7 μM in 143B cells. The expression of ferroptosis-related genes was examined in 143B and U2OS cells. **(A)** Analysis of ferroptosis-related gene expression in 143B and U2OS cells. **(B)** Analysis of ferroptosis-related gene expression in 143B cells after co-treatment with Fer-1 and lenvatinib. **(C)** Analysis of ferroptosis-related gene expression in U2OS cells after co-treatment with Fer-1 and lenvatinib. Data are presented as the mean ± standard deviation (SD) (n = 3). ns, not significant; *p < 0.05, **p < 0.01, ***p < 0.001.

The results showed that, compared with the control group, lenvatinib treatment upregulated the expression of the tumor suppressor p53 and the ferroptosis-associated marker PTGS2, while downregulating the expression of GPX4 and xCT (**Figure 5A**). These findings suggest that lenvatinib-induced ferroptosis-related changes are accompanied by alterations in ferroptosis-related gene expression.

To determine whether the ferroptosis inhibitor Fer-1 could reverse the ferroptosis-related gene expression changes induced by lenvatinib in 143B and U2OS cells, qRT-PCR was performed. The results showed that, compared with the lenvatinib-treated group, the Fer-1 plus lenvatinib co-treatment group exhibited decreased expression of p53 and PTGS2, but increased expression of GPX4 and xCT. In contrast, Fer-1 alone had no significant effect on the expression of ferroptosis-related genes in osteosarcoma cells compared with the control group (**Figures 5B, C**). These findings indicate that Fer-1 reversed the lenvatinib-induced changes in ferroptosis-related gene expression in osteosarcoma cells.

#### 3.5.2 Lenvatinib regulated ferroptosis-related protein expression in a concentration-dependent manner, and these effects were reversed by Fer-1

Next, Western blotting was performed to examine the expression of ferroptosis-related proteins, including p-STAT3, STAT3, p53, GPX4, and xCT, in 143B and U2OS cells after treatment with different concentrations of lenvatinib.

**Figure 6:**
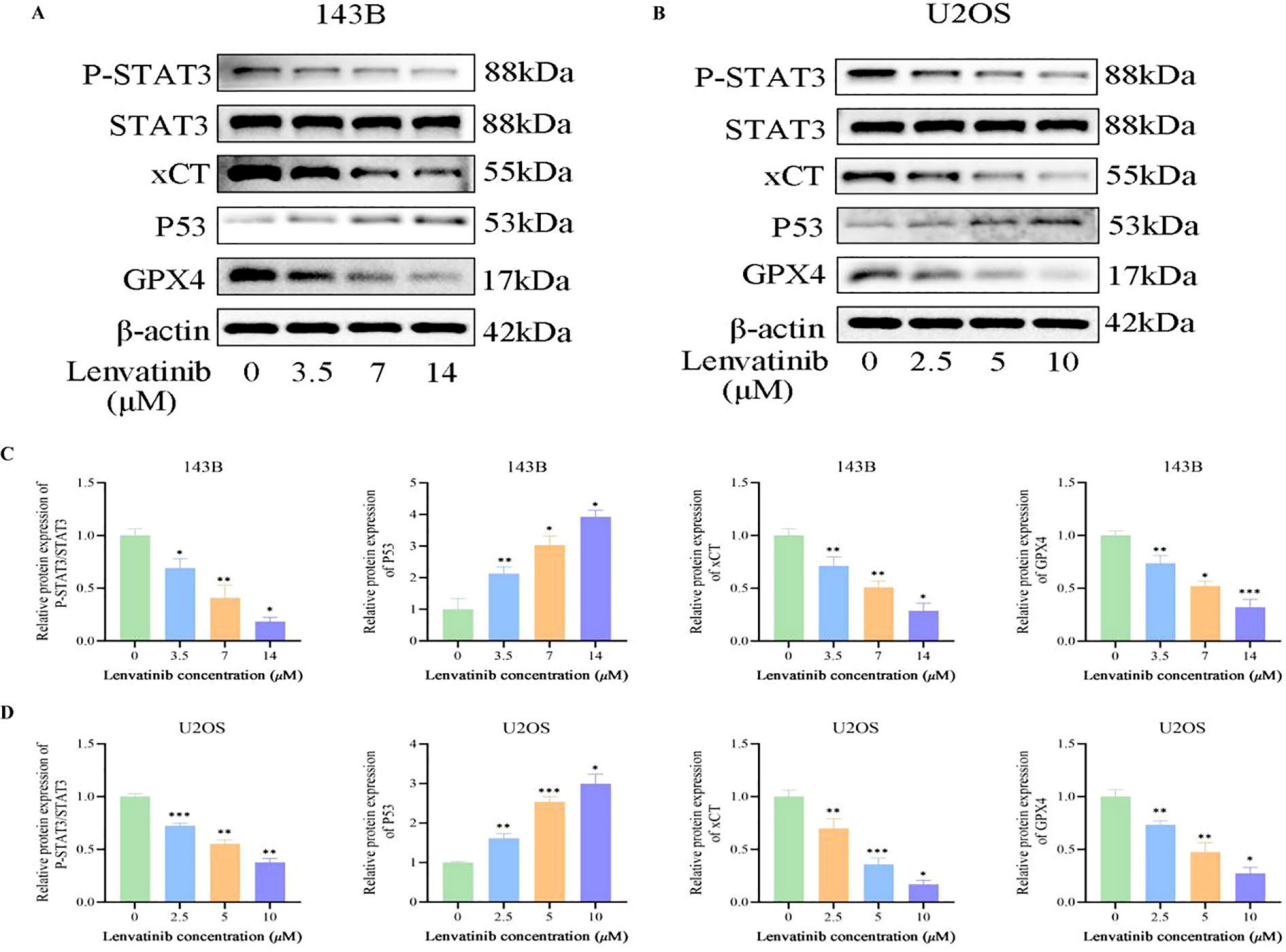
Lenvatinib regulated ferroptosis-related protein expression in osteosarcoma cells. Cells were treated with lenvatinib for 24 h. The treatment concentrations were 0, 2.5, 5, and 10 μM for U2OS cells, and 0, 3.5, 7, and 14 μM for 143B cells. The expression levels of ferroptosis-related proteins in 143B and U2OS cells were detected by Western blotting. **(A)** Concentration-dependent changes in ferroptosis-related protein expression in 143B cells. **(B)** Concentration-dependent changes in ferroptosis-related protein expression in U2OS cells. **(C)** Quantitative analysis of protein expression differences in 143B cells. **(D)** Quantitative analysis of protein expression differences in U2OS cells. Data are presented as the mean ± standard deviation (SD) (n = 3). *p < 0.05, **p < 0.01, ***p < 0.001.

The results showed that total STAT3 expression was not markedly altered with increasing lenvatinib concentrations. In contrast, the expression levels of p-STAT3, GPX4, and xCT were significantly decreased, whereas p53 expression was significantly increased in a concentration-dependent manner (**Figures 6A, B**). Quantitative analysis further showed that the p-STAT3/STAT3 ratio was significantly reduced after lenvatinib treatment (**Figures 6C, D**). These results suggest that lenvatinib-induced ferroptosis-related changes are associated with suppression of STAT3 phosphorylation.

**Figure 7:**
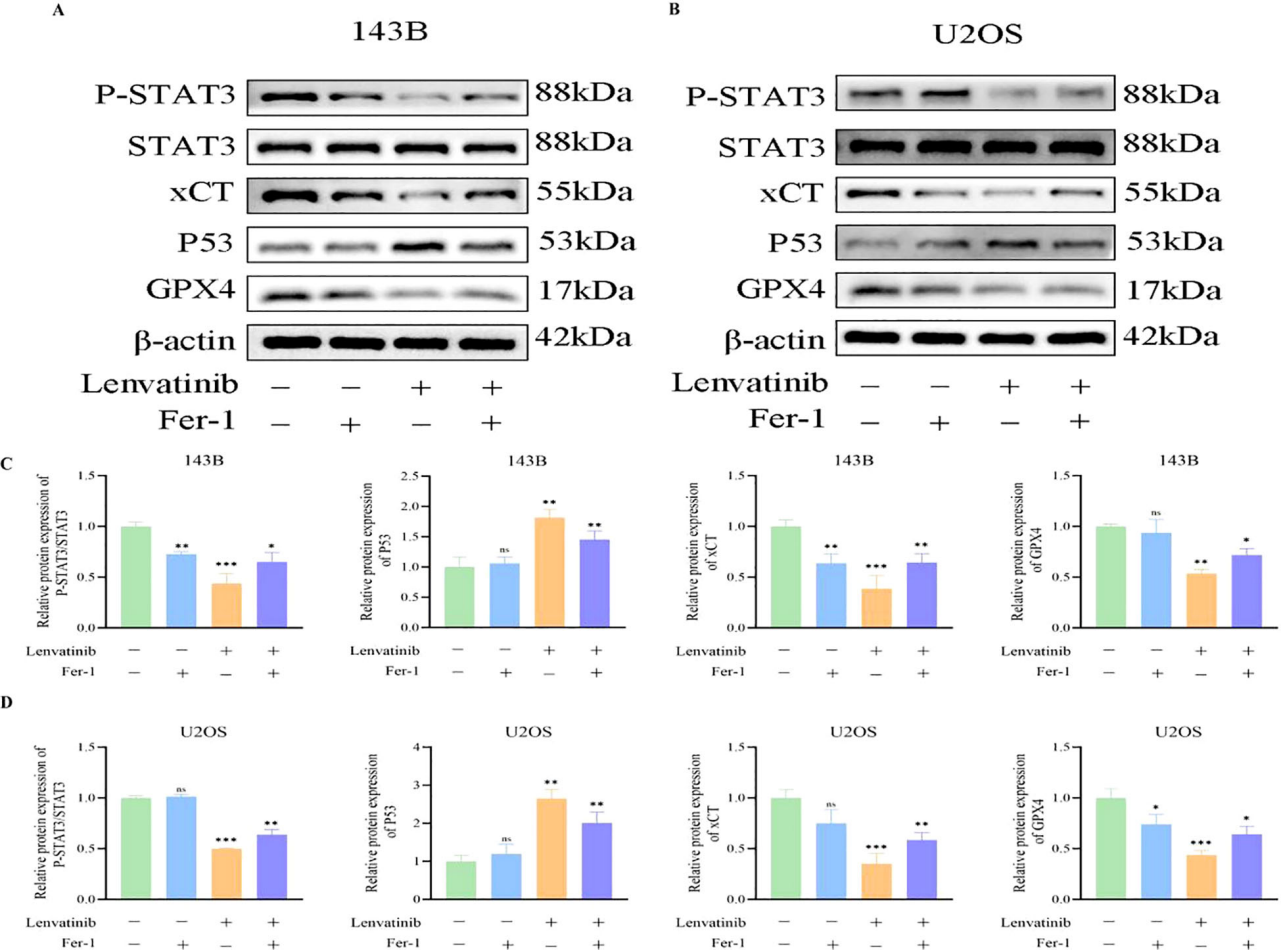
Fer-1 reversed lenvatinib-induced changes in ferroptosis-related protein expression in osteosarcoma cells. Cells were pretreated with Fer-1 for 1 h and then co-treated with lenvatinib for 24 h. The concentration of Fer-1 was 5 μM. Lenvatinib was used at 5 μM in U2OS cells and 7 μM in 143B cells. **(A)** Changes in protein expression in 143B cells after co-treatment with Fer-1 and lenvatinib. **(B)** Changes in protein expression in U2OS cells after co-treatment with Fer-1 and lenvatinib. **(C)** Quantitative analysis of protein expression in 143B cells after co-treatment with Fer-1 and lenvatinib. **(D)** Quantitative analysis of protein expression in U2OS cells after co-treatment with Fer-1 and lenvatinib. Data are presented as the mean ± standard deviation (SD) (n = 3). ns, not significant; *p < 0.05, **p < 0.01, ***p < 0.001.

To determine whether the ferroptosis inhibitor Fer-1 could reverse lenvatinib-induced changes in ferroptosis-related protein expression in 143B and U2OS cells, Western blotting was performed. The results showed that, compared with the lenvatinib-treated group, the Fer-1 plus lenvatinib co-treatment group showed no marked change in total STAT3 expression, whereas p53 expression was decreased and the expression levels of p-STAT3, GPX4, and xCT were increased (**Figures 7A, B**). In addition, the p-STAT3/STAT3 ratio showed a statistically significant change (**Figures 7C, D**), further indicating that lenvatinib-induced ferroptosis in osteosarcoma cells was associated with inhibition of STAT3 phosphorylation.

Together, the ferroptosis inhibition experiments suggest that lenvatinib-induced ferroptosis-related changes are associated with reduced STAT3 phosphorylation, increased p53 expression, and decreased xCT expression.

#### 3.5.3 Lenvatinib treatment was associated with increased p53 expression and reduced xCT expression in osteosarcoma cells

**Figure 8:**
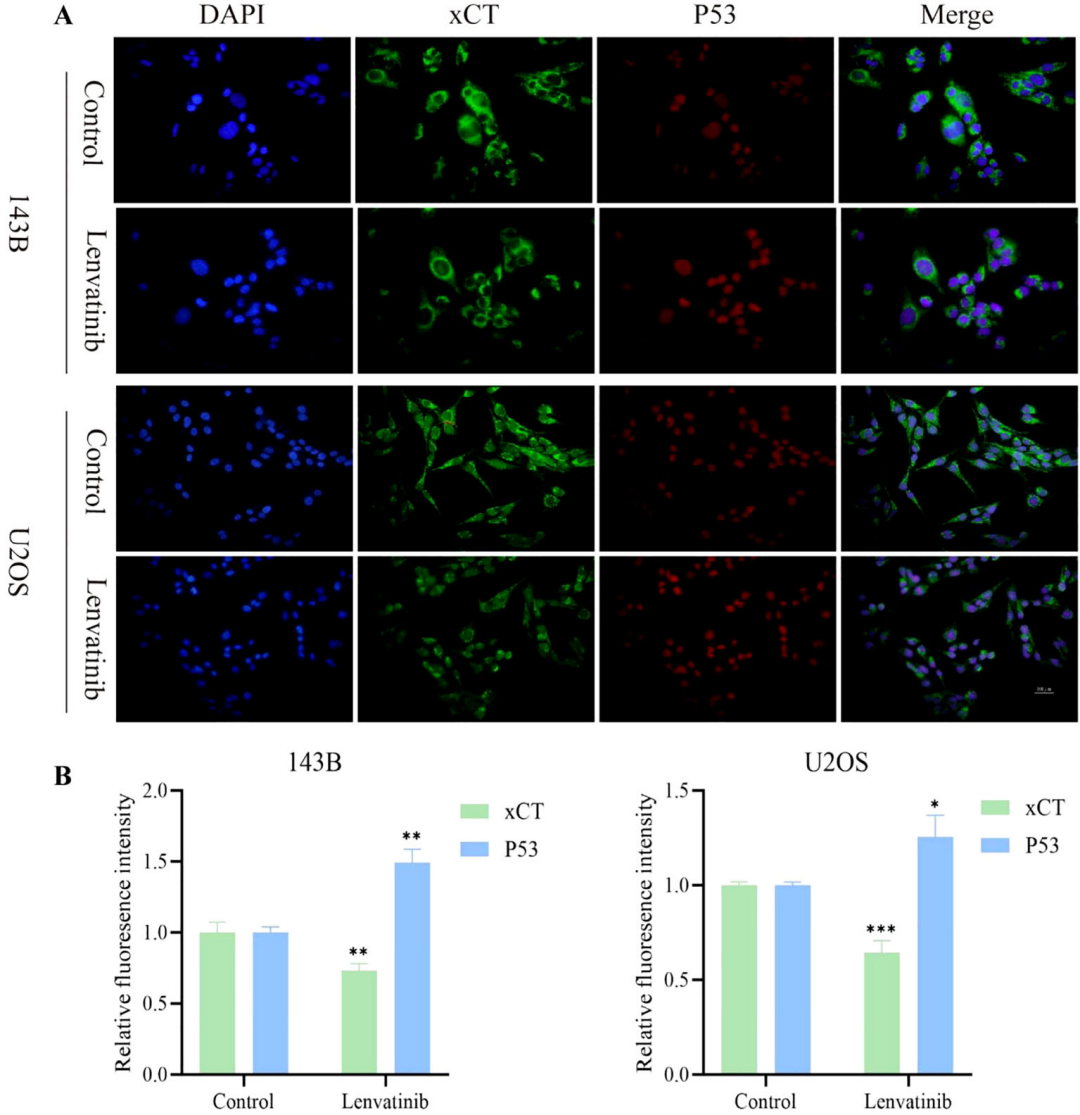
Lenvatinib treatment was associated with increased p53 expression and reduced xCT expression in osteosarcoma cells. Cells were treated with lenvatinib for 24 h. The treatment concentrations were 5 μM for U2OS cells and 7 μM for 143B cells. **(A)** Changes in p53 and xCT fluorescence intensity were detected by immunofluorescence staining. Scale bar = 100 μm. **(B)** Quantitative analysis of p53 and xCT fluorescence intensity in 143B and U2OS cells. Data are presented as the mean ± standard deviation (SD) (n = 3). *p < 0.05, **p < 0.01, ***p < 0.001.

Finally, to further examine the relationship between p53 expression and xCT expression after lenvatinib treatment, double immunofluorescence staining was performed in 143B and U2OS cells. The results showed that, compared with the control group, p53 fluorescence intensity was increased and was predominantly localized in the nucleus, whereas xCT fluorescence intensity was reduced and was mainly localized in the cytoplasm (**Figures 8A, B**). These findings were consistent with the changes in p53 and xCT expression observed by qRT-PCR and Western blotting.

Together, these results support a regulatory association between increased p53 expression and reduced xCT expression in osteosarcoma cells. In addition, previous studies have documented a regulatory relationship between p-STAT3 and p53, which is consistent with our Western blotting results. Accordingly, our data suggest that lenvatinib-induced ferroptosis-related changes may involve reduced STAT3 phosphorylation, increased p53 expression, and associated downregulation of xCT expression; however, the causal sequence among these molecules requires further validation.

### 3.6 xCT upregulation attenuated lenvatinib-induced ferroptosis-related changes and promoted migration and invasion in osteosarcoma cells

**Figure 9:**
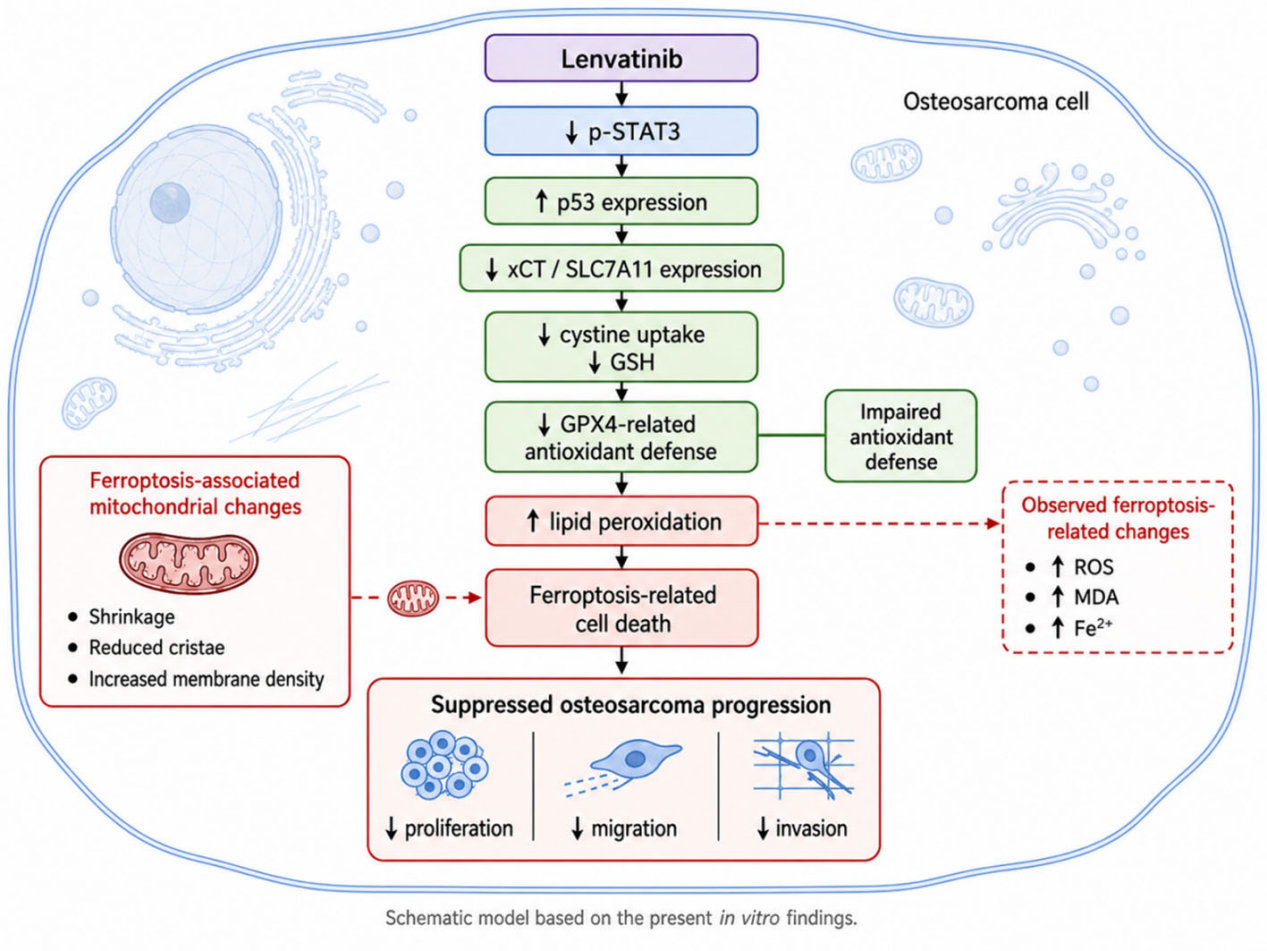
Proposed mechanism of lenvatinib-induced ferroptosis-related changes in osteosarcoma cells. Lenvatinib treatment in osteosarcoma cells was associated with decreased p-STAT3, increased p53, and reduced xCT/SLC7A11 expression, leading to decreased cystine uptake, reduced GSH, impaired GPX4 antioxidant defense, increased lipid peroxidation, and ferroptosis-related cell death. Observed ferroptosis-associated changes included elevated ROS, MDA, Fe^2+^ and mitochondrial alterations. These events were accompanied by reduced proliferation, migration, and invasion. The diagram represents a schematic model based on *in vitro* findings and summarizes the proposed pathway rather than definitive causality.

It is well established that the system Xc^-^ transporter plays a critical role in suppressing ferroptosis, with its light-chain subunit xCT mediating cystine uptake and glutamate export, thereby supporting GSH synthesis. To further evaluate the involvement of xCT in lenvatinib-induced ferroptosis-related changes, stable xCT-overexpressing cell models were established for subsequent validation experiments. A proposed mechanism summarizing our *in vitro* findings is shown in **Figure 9**.

#### 3.6.1 xCT upregulation attenuated lenvatinib-induced cell death in osteosarcoma cells

To investigate the effect of lenvatinib on 143B and U2OS cell proliferation after xCT upregulation, cell viability was assessed using the CCK-8 assay following lenvatinib treatment in xCT-overexpressing osteosarcoma cells.

**Figure 10:**
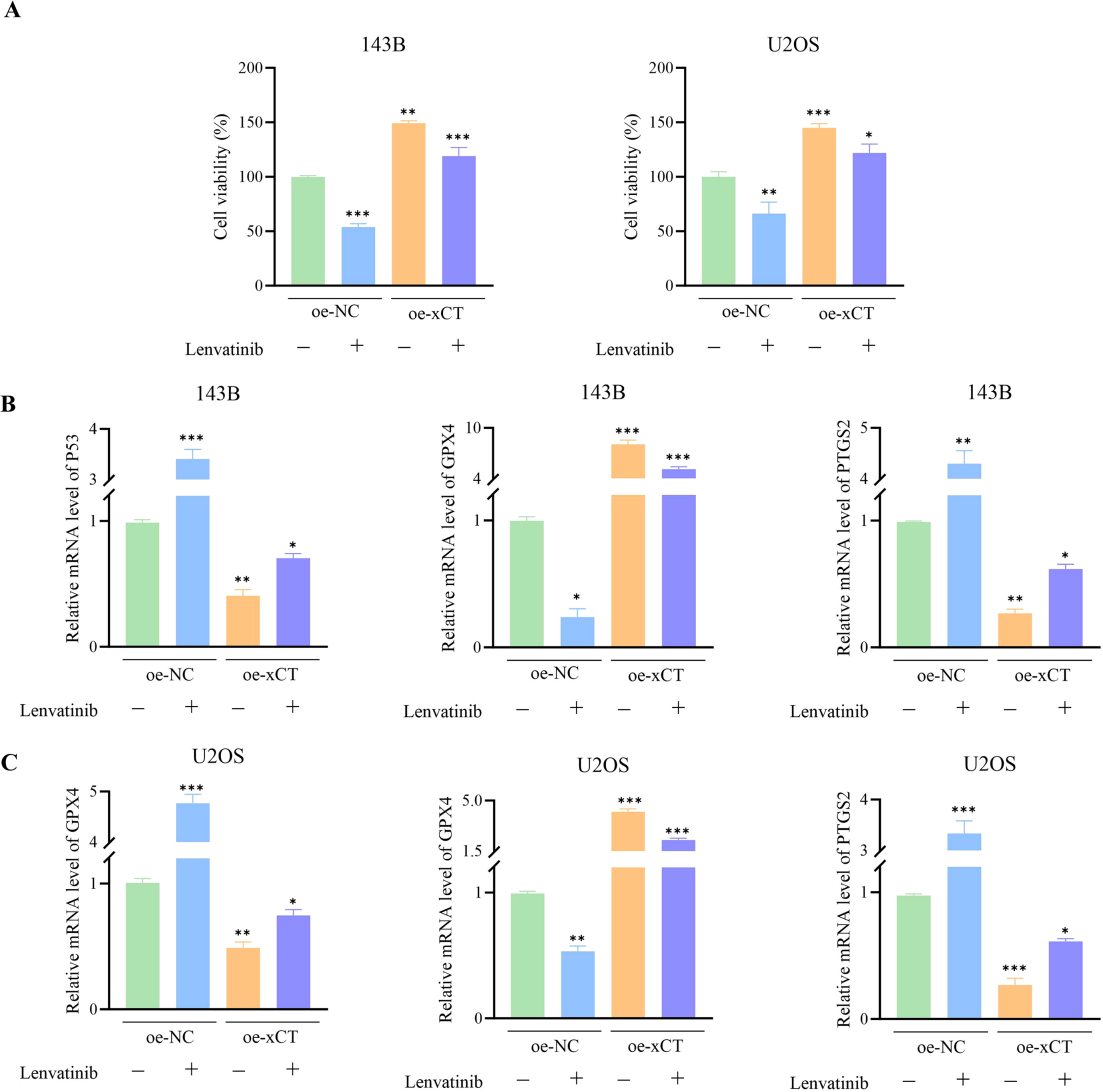
xCT upregulation attenuated lenvatinib-induced cell death and ferroptosis-related gene expression changes in osteosarcoma cells. Cells were treated with lenvatinib for 24 h. The concentrations of lenvatinib were 5 μM for U2OS cells and 7 μM for 143B cells. **(A)** xCT upregulation attenuated lenvatinib-induced cell death in 143B and U2OS cells. **(B)** Quantitative analysis of ferroptosis-related gene expression in 143B cells after xCT upregulation. **(C)** Quantitative analysis of ferroptosis-related gene expression in U2OS cells after xCT upregulation. Data are presented as the mean ± standard deviation (SD) (n = 3). *p < 0.05, **p < 0.01, ***p < 0.001.

The results showed that, compared with the overexpression negative control group, xCT upregulation significantly promoted osteosarcoma cell proliferation. In contrast, compared with the untreated group, lenvatinib treatment markedly inhibited osteosarcoma cell proliferation (**Figure 10A**). These findings indicate that xCT upregulation enhanced osteosarcoma cell viability and partially attenuated lenvatinib-induced growth inhibition.

#### 3.6.2 xCT Upregulation Attenuated Lenvatinib-Induced Changes in Ferroptosis-Related Gene Expression in Osteosarcoma Cells

To investigate the effect of xCT upregulation on lenvatinib-induced ferroptosis-related gene expression in 143B and U2OS cells, qRT-PCR was performed. The results showed that, compared with the oe-NC group, the oe-xCT group exhibited significantly reduced expression of p53 and PTGS2, but increased expression of GPX4. In contrast, compared with the untreated group, lenvatinib treatment markedly increased the expression of p53 and PTGS2, while decreasing GPX4 expression (**Figures 10B, C**).

These findings indicate that xCT upregulation attenuated the lenvatinib-induced changes in ferroptosis-related gene expression in osteosarcoma cells.

#### 3.6.3 xCT Upregulation attenuated lenvatinib-induced changes in ferroptosis-related protein expression in osteosarcoma cells

**Figure 11:**
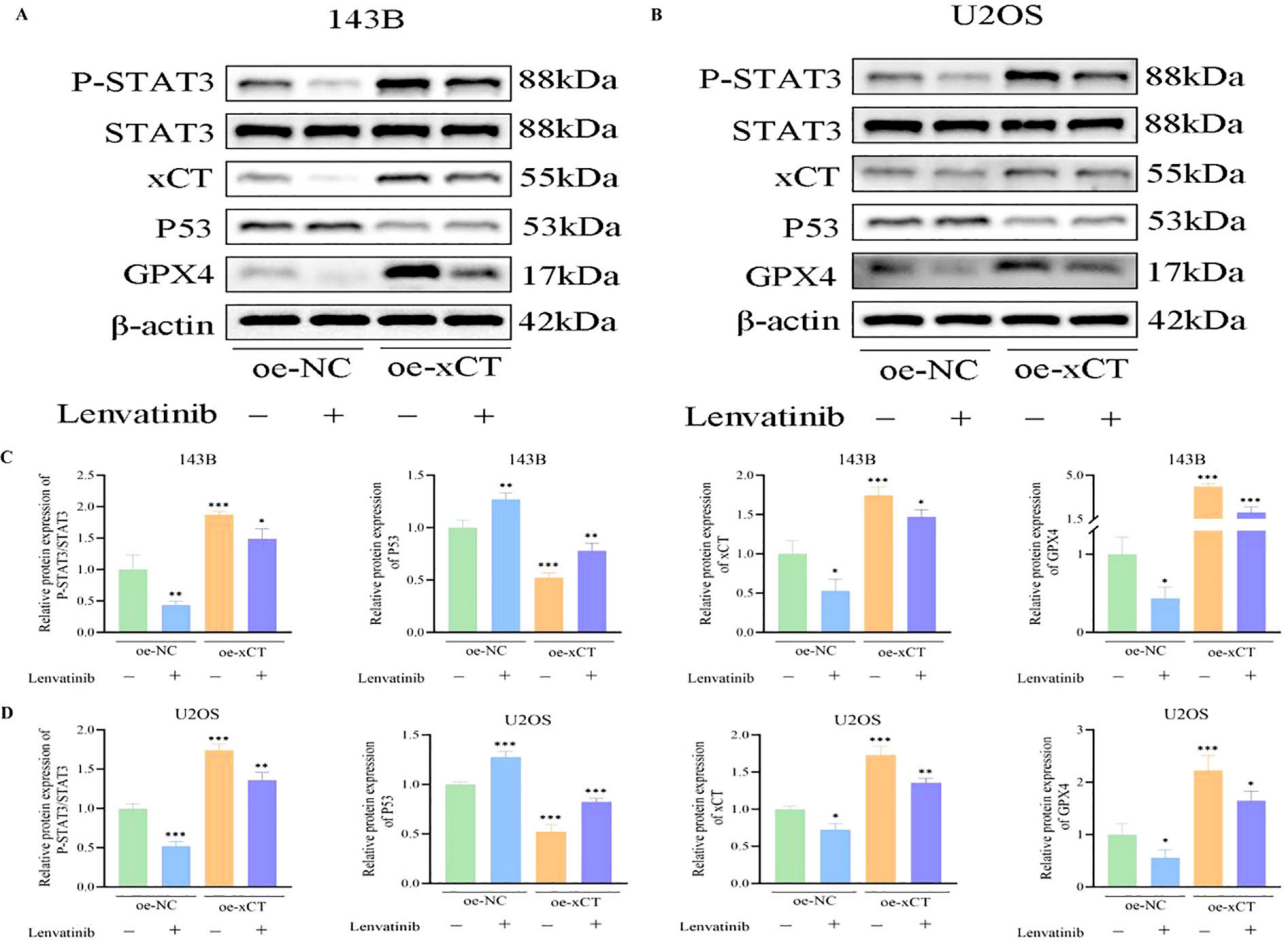
xCT upregulation attenuated lenvatinib-induced changes in ferroptosis-related protein expression in osteosarcoma cells. Cells were treated with lenvatinib for 24 h. The concentrations of lenvatinib were 5 μM for U2OS cells and 7 μM for 143B cells. **(A)** Changes in ferroptosis-related protein expression in 143B cells after xCT upregulation. **(B)** Changes in ferroptosis-related protein expression in U2OS cells after xCT upregulation. **(C)** Quantitative analysis of ferroptosis-related protein expression in 143B cells after xCT upregulation. **(D)** Quantitative analysis of ferroptosis-related protein expression in U2OS cells after xCT upregulation. Data are presented as the mean ± standard deviation (SD) (n = 3). *p < 0.05, **p < 0.01, ***p < 0.001.

To investigate the effect of xCT upregulation on lenvatinib-induced changes in ferroptosis-related protein expression in 143B and U2OS cells, Western blotting was performed. The results showed that, compared with the oe-NC group, the oe-xCT group exhibited no obvious change in total STAT3 expression, whereas p53 expression was decreased and the expression levels of p-STAT3, GPX4, and xCT were increased. In contrast, compared with the untreated group, lenvatinib treatment still did not significantly alter total STAT3 expression, but increased p53 expression and decreased the expression levels of p-STAT3, GPX4, and xCT (**Figures 11A, B**).

Moreover, the p-STAT3/STAT3 ratio showed a statistically significant difference (**Figures 11C, D**), further supporting an association between lenvatinib-induced ferroptosis-related changes and reduced STAT3 phosphorylation in 143B and U2OS cells.

#### 3.6.4 xCT upregulation attenuated the inhibitory effects of lenvatinib on osteosarcoma cell migration and invasion

Aberrant activation of cell migration and invasion is a key feature of malignant tumors. To investigate the effect of xCT upregulation on lenvatinib-mediated inhibition of migration and invasion in 143B and U2OS cells, wound-healing and Transwell assays were performed.

**Figure 12:**
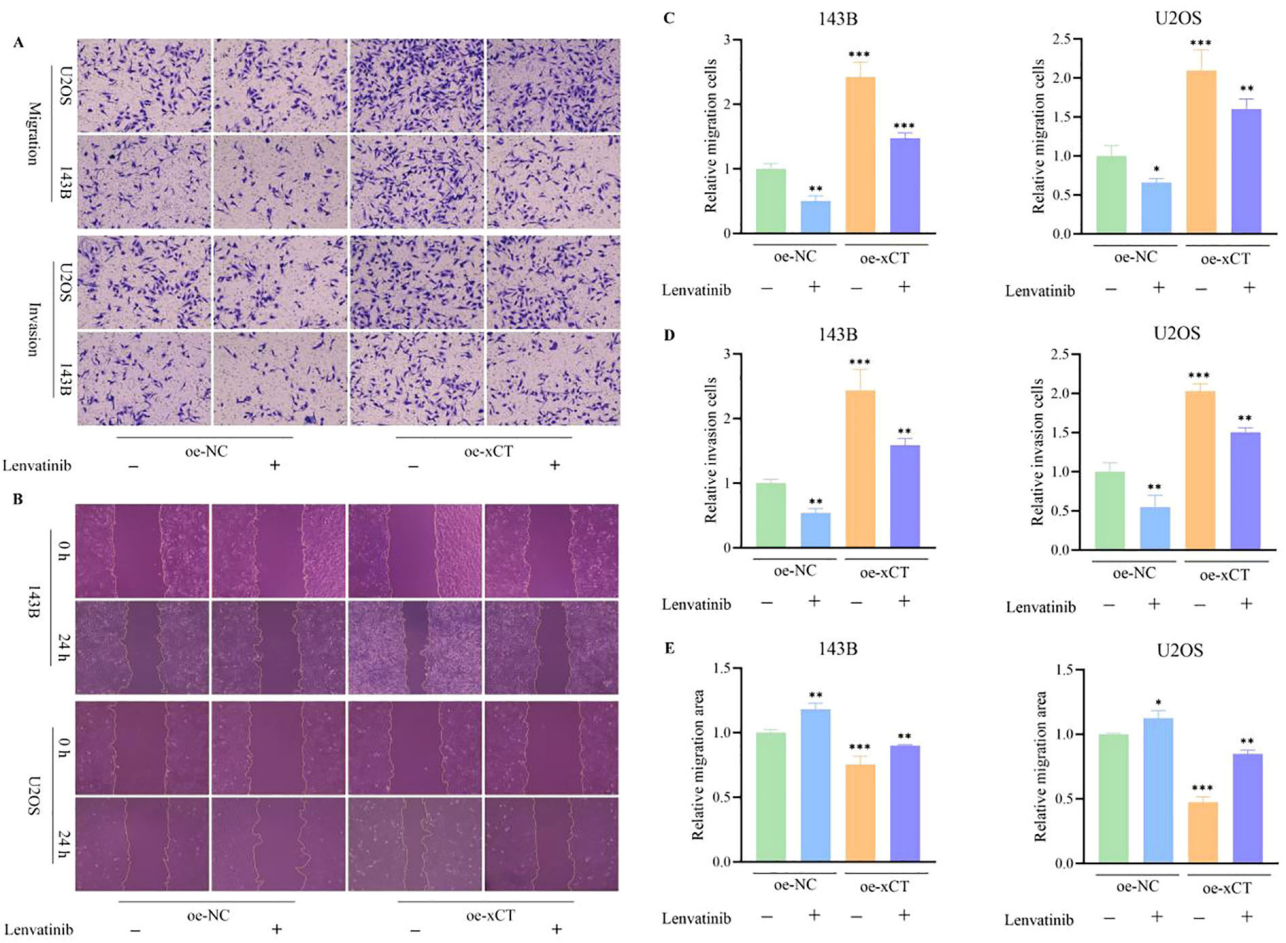
xCT upregulation attenuated the inhibitory effects of lenvatinib on osteosarcoma cell migration and invasion. Cells were treated with lenvatinib for 24 h. The concentrations of lenvatinib were 5 μM for U2OS cells and 7 μM for 143B cells. **(A)** Transwell assays were performed to evaluate the effects of xCT upregulation on lenvatinib-induced changes in the migratory and invasive capacities of osteosarcoma cells. **(B)** Wound-healing assays were performed to evaluate the effects of xCT upregulation on lenvatinib-induced changes in the migratory ability of osteosarcoma cells. **(C)** Quantitative analysis of the number of migrated U2OS and 143B cells after xCT upregulation. **(D)** Quantitative analysis of the number of invaded U2OS and 143B cells after xCT upregulation. **(E)** Quantitative analysis of wound closure area in U2OS and 143B cells after xCT upregulation. Data are presented as the mean ± standard deviation (SD) (n = 3). *p < 0.05, **p < 0.01, ***p < 0.001.

The Transwell assay showed that, compared with the oe-NC group, xCT upregulation significantly enhanced the migratory and invasive abilities of osteosarcoma cells. In contrast, compared with the untreated group, lenvatinib treatment markedly suppressed osteosarcoma cell migration and invasion (**Figures 12A, C, D**).

Consistently, the wound-healing assay showed that, compared with the oe-NC group, xCT upregulation significantly promoted osteosarcoma cell migration, whereas lenvatinib treatment markedly inhibited cell migration compared with the untreated group (**Figures 12B, E**).

## 4 Discussion

The present study suggests that lenvatinib induces ferroptosis-related changes in osteosarcoma cells and suppresses osteosarcoma cell proliferation, migration, and invasion *in vitro*. Mechanistically, these effects were accompanied by reduced STAT3 phosphorylation, increased p53 expression, decreased xCT expression, and impaired GPX4-related ferroptosis defense. These findings provide preliminary evidence for a previously underrecognized mechanism of lenvatinib action in osteosarcoma and support further investigation of ferroptosis-related therapeutic strategies.

Osteosarcoma is the most common primary malignant bone tumor and is characterized by aggressive biological behavior and poor prognosis (23). Surgery combined with neoadjuvant chemotherapy remains the standard first-line treatment strategy for osteosarcoma. However, both limb-salvage surgery and amputation are associated with substantial clinical challenges, including postoperative recurrence, distant metastasis, extensive surgical trauma, and a marked decline in quality of life (24, 25). Moreover, although neoadjuvant chemotherapy has improved survival in some patients, its clinical efficacy is still limited by drug resistance, severe chemotherapy-related adverse reactions, and poor treatment tolerance (26–28). Therefore, identifying new therapeutic strategies is essential for improving the treatment efficacy and prognosis of patients with osteosarcoma.

Iron is an indispensable trace element in the human body and plays a critical role in growth, development, oxygen transport, and oxygen storage. In 2012, Dixon et al. (29) first proposed the concept of ferroptosis and demonstrated that erastin induced a distinct form of iron-dependent cell death closely associated with inhibition of cystine uptake. Ferroptosis differs from apoptosis, pyroptosis, and other forms of regulated cell death in terms of morphology, biochemistry, and genetic regulation. Mechanistically, ferroptosis depends on intracellular iron accumulation and is accompanied by ROS accumulation, lipid peroxidation, mitochondrial shrinkage, loss of mitochondrial cristae, and increased mitochondrial membrane density. As a newly recognized form of regulated cell death, ferroptosis is closely linked to alterations in the cellular microenvironment, which is also a key determinant of tumor initiation and progression. These observations indicate a close mechanistic relationship between ferroptosis and tumor biology. Accordingly, inducing ferroptosis may represent an effective therapeutic strategy for osteosarcoma and may provide a new direction for the development of anti-osteosarcoma therapies.

Lenvatinib is an antitumor agent that has been used for refractory, recurrent, or unresectable osteosarcoma (30, 31). In addition, lenvatinib combined with PD-1 blockade has been reported to improve therapeutic outcomes in patients with metastatic osteosarcoma (32). Previous studies have also shown that lenvatinib can induce apoptosis in osteosarcoma cells by inhibiting protein kinase B signaling (33). Liang et al. (22) reported that lenvatinib-induced hypertension is associated with ferroptosis in endothelial cells and that ferroptosis inhibition can alleviate endothelial dysfunction and reduce blood pressure. Another study demonstrated that, in hepatocellular carcinoma cells, lenvatinib inhibits xCT expression through suppression of FGFR4, thereby promoting lipid ROS accumulation and ferroptosis; furthermore, inhibition of activated Nrf2 enhanced the sensitivity of hepatocellular carcinoma cells to lenvatinib (21). However, whether lenvatinib exerts antitumor effects in osteosarcoma by inducing ferroptosis remains unclear. Therefore, the present study investigated lenvatinib as a potential ferroptosis-inducing agent in osteosarcoma cells.

Ferroptosis is mainly counteracted by three defense systems mediated by DHODH, FSP1, and GPX4. DHODH primarily functions in mitochondria to protect cells against ferroptosis. Mitochondria are essential sites for the tricarboxylic acid cycle and *de novo* pyrimidine synthesis. DHODH is located in the inner mitochondrial membrane and plays an important role in pyrimidine biosynthesis, with CoQ10 serving as its electron acceptor (9). Through DHODH, the oxidation of dihydroorotate to orotate is coupled with the reduction of ubiquinone to ubiquinol, forming the DHODH-CoQH2 axis, which acts similarly to a radical-trapping antioxidant and suppresses mitochondrial lipid peroxidation, thereby inhibiting ferroptosis (34).

FSP1 is a flavoprotein also known as apoptosis-inducing factor mitochondria-associated protein 2 because of its high homology with apoptosis-inducing factor (35, 36). FSP1 is a potent anti-ferroptotic factor that translocates to the plasma membrane after myristoylation. At this site, FSP1 functions as an NADH-dependent oxidoreductase and reduces CoQ10 to CoQH2 by consuming NADH/NADPH. CoQH2 generated by FSP1 serves as a lipophilic radical-trapping antioxidant, thereby suppressing lipid peroxide accumulation and inhibiting ferroptosis. In multiple tumor cell types, high FSP1 expression is negatively associated with ferroptosis sensitivity (8, 37). The anti-ferroptotic function of FSP1 depends on the mevalonate pathway, which begins with acetyl-CoA and ultimately contributes to the synthesis of cholesterol and other products, including isopentenyl pyrophosphate, CoQ10, and squalene (38).

Among these pathways, the GPX4-dependent pathway is the most classical and central mechanism regulating ferroptosis. It is mediated by both an intrinsic regulatory pathway involving GPX4 and an extrinsic regulatory pathway involving system Xc^-^, in which the light-chain subunit xCT plays a key anti-ferroptotic role (13). Polyunsaturated fatty acids are essential substrates for phospholipid hydroperoxide formation. GPX4 uses electrons provided by GSH and its own selenocysteine residue to reduce phospholipid hydroperoxides to phospholipid alcohols and hydrogen peroxide to water. In this way, GPX4 interrupts lipid peroxidation driven by ROS accumulation, which is a critical step in ferroptotic cell death (39, 40). Because GPX4 can regulate ferroptosis in both mitochondria and the cytoplasm, and because the GPX4-dependent pathway is central to ferroptosis regulation, this study focused on this ferroptosis defense pathway.

In the present study, CCK-8 assays showed that lenvatinib inhibited osteosarcoma cell proliferation in a concentration-dependent manner. Under the same experimental conditions, lenvatinib had no obvious effect on the proliferation of hFOB1.19 osteoblasts, suggesting a relatively selective inhibitory effect on osteosarcoma cells. With increasing treatment duration, the inhibitory effect of lenvatinib became more evident in 143B and U2OS cells, whereas this pattern was not observed in MG63 cells. Based on the IC50 values, lenvatinib showed stronger anti-proliferative effects in 143B and U2OS cells than in MG63 cells. In addition, the inhibitory effects observed in 143B and U2OS cells after 48 and 72 h of treatment were not superior to those observed after 24 h. Therefore, 143B and U2OS cells treated for 24 h were selected for subsequent experiments, with lenvatinib concentrations of 7 μM and 5 μM, respectively.

To determine whether lenvatinib altered mitochondrial morphology in osteosarcoma cells, TEM was performed. The results showed typical ferroptosis-associated mitochondrial alterations in 143B and U2OS cells, including reduced mitochondrial volume, increased mitochondrial membrane density, and decreased or absent mitochondrial cristae. In contrast, typical morphological features of other forms of cell death, such as apoptotic bodies in apoptosis, organelle swelling in necroptosis, or double-membrane autolysosomes in autophagy, were not observed (41, 42). These ultrastructural findings support the involvement of ferroptosis-related cell death in lenvatinib-treated osteosarcoma cells.

Previous studies have confirmed that lenvatinib can induce ferroptosis in hepatocellular carcinoma by inhibiting FGFR4 (21). However, whether lenvatinib can induce ferroptosis-related phenotypic changes in osteosarcoma has not been fully clarified. Therefore, ferroptosis-related phenotypes were evaluated in the present study. After 24 h of lenvatinib treatment, flow cytometry and biochemical assays showed that intracellular Fe^2+^ levels, ROS fluorescence intensity, and MDA levels were significantly increased in 143B and U2OS cells in a concentration-dependent manner. Conversely, intracellular reduced GSH levels decreased progressively with increasing lenvatinib concentration. Taken together, these findings suggest that lenvatinib induces ferroptosis-related changes in osteosarcoma cells and that this effect is positively associated with drug concentration.

xCT is a key component of system Xc^-^, which regulates GSH synthesis and participates in ferroptosis control through the GSH-GPX4 axis. Ferroptosis inhibitors associated with xCT-related pathways include liproxstatin-1, ferrostatin-1, and desferrioxamine. Fer-1 can inhibit iron accumulation and also act as a scavenger of lipid peroxyl radicals. During ferroptosis, excessive Fe^2+^ accumulates in the labile iron pool and generates large amounts of hydroxyl radicals through the Fenton and Haber-Weiss reactions. This leads to a sharp increase in intracellular ROS, triggers lipid peroxidation, and ultimately induces ferroptotic cell death. Because Fer-1 can interfere not only with the GSH-GPX4 axis but also with iron metabolism and lipid peroxidation, it was selected for ferroptosis inhibition experiments in this study.

Previous studies have shown that restoration of cell viability by ferroptosis inhibitors is an important feature supporting ferroptotic cell death (43–45). In the present study, CCK-8 assays showed that, compared with lenvatinib treatment alone, co-treatment with Fer-1 and lenvatinib markedly restored osteosarcoma cell viability. Fer-1 alone had no obvious effect on osteosarcoma cell viability compared with the control group. To further verify whether ferroptosis inhibition could reverse lenvatinib-induced phenotypic changes, ferroptosis-related markers were examined. Compared with the lenvatinib group, the Fer-1 plus lenvatinib co-treatment group showed reduced ROS fluorescence intensity, decreased MDA and Fe^2+^ levels, and increased reduced GSH levels. In contrast, Fer-1 alone did not significantly alter these ferroptosis-related phenotypes compared with the control group. These results demonstrate that Fer-1 reverses lenvatinib-induced ferroptosis-related phenotypic alterations in osteosarcoma cells, further supporting the involvement of ferroptosis-related cell death in the response to lenvatinib.

The GPX4-dependent regulation of ferroptosis involves two major mechanisms: direct inhibition of GPX4 and indirect inhibition of system Xc^-^ (7, 46). In system Xc^-^, the light-chain subunit xCT mediates cystine uptake and glutamate export, thereby supporting GSH synthesis (12, 13). Previous studies have shown that ferroptosis is accompanied by upregulation of the ferroptosis-associated gene PTGS2 and downregulation of GPX4 and xCT (18, 47). Zhang et al. (48) reported that oridonin effectively induced ferroptosis in osteosarcoma cells by suppressing GPX4 and xCT expression. Tirapazamine has also been reported to induce ferroptosis in osteosarcoma cells by inhibiting GPX4 and xCT, and xCT upregulation reversed tirapazamine-induced changes in cell viability, ferroptosis-related phenotypes, and protein expression (20).

In this study, qRT-PCR was first performed to determine whether lenvatinib regulated PTGS2, GPX4, and xCT expression in 143B and U2OS cells. Compared with the control group, PTGS2 expression was significantly increased, whereas GPX4 and xCT expression was decreased. Western blotting further showed that the protein expression patterns of GPX4 and xCT were consistent with the qRT-PCR results. To determine whether Fer-1 could reverse these expression changes, qRT-PCR was performed after Fer-1 and lenvatinib co-treatment. Compared with the lenvatinib group, the co-treatment group showed decreased PTGS2 expression and increased GPX4 and xCT expression, whereas Fer-1 alone had no obvious effect on ferroptosis-related gene expression compared with the control group. Western blotting confirmed that GPX4 and xCT protein expression changes were consistent with the mRNA results. These findings suggest that lenvatinib may impair the GPX4-dependent ferroptosis defense pathway by reducing GPX4 and xCT expression in osteosarcoma cells.

Activation of phosphorylated signal transducer and activator of transcription 3 is critical for tumor initiation and progression and is also closely associated with ferroptosis. Previous studies have shown that inhibition of p-STAT3 activity induces ferroptosis and significantly enhances cisplatin sensitivity in osteosarcoma cells. Treatment with a STAT3 inhibitor attenuated cisplatin-induced p-STAT3 upregulation and increased cisplatin sensitivity in resistant cells (49). Li et al. (39) reported that FANCD2 induces ferroptosis in osteosarcoma by suppressing p-STAT3. Other studies have shown that bavachin induces ferroptosis in osteosarcoma cells by inhibiting p-STAT3 activity, and STAT3 upregulation reverses bavachin-induced cell death and ferroptosis-related phenotypic changes (47).

Based on these findings, we hypothesized that lenvatinib may also induce ferroptosis in osteosarcoma cells by inhibiting p-STAT3 activity. Western blotting showed that total STAT3 expression did not change significantly with increasing lenvatinib concentration, whereas p-STAT3 expression was markedly decreased. The p-STAT3/STAT3 ratio was significantly reduced, indicating that lenvatinib-mediated ferroptosis in osteosarcoma cells was associated with inhibition of STAT3 phosphorylation in a concentration-dependent manner. To further examine this association, p-STAT3 protein expression was examined in the ferroptosis inhibition experiments. Compared with the lenvatinib group, the Fer-1 plus lenvatinib co-treatment group showed no obvious change in total STAT3 expression but exhibited increased p-STAT3 expression, with a significant alteration in the p-STAT3/STAT3 ratio. These results further support an association between lenvatinib-induced ferroptosis-related changes and suppression of STAT3 phosphorylation.

p53 is an upstream regulator of xCT and plays an important role in ferroptosis. Liu et al. (40) reported that gambogic acid induces ferroptosis in osteosarcoma cells by activating p53. However, other studies have shown that p53 can also promote FSP1 expression in osteosarcoma cells, thereby inhibiting ferroptosis (50). These findings suggest that p53 has context-dependent and dual regulatory functions in osteosarcoma ferroptosis. Therefore, determining how p53 functions under specific therapeutic conditions is essential.

To clarify whether p53 promoted or inhibited lenvatinib-induced ferroptosis in osteosarcoma cells, p53 expression was examined by qRT-PCR. Compared with the control group, p53 expression was upregulated after lenvatinib treatment. Western blotting confirmed that p53 protein expression changed consistently with the mRNA results. In ferroptosis inhibition experiments, qRT-PCR showed that p53 expression was decreased in the Fer-1 plus lenvatinib co-treatment group compared with the lenvatinib group, and the corresponding protein expression pattern was consistent with this result. Double immunofluorescence staining further showed that p53 fluorescence intensity was increased and mainly localized in the nucleus after lenvatinib treatment, whereas xCT fluorescence intensity was decreased and mainly localized in the cytoplasm. Based on these gene, protein, and fluorescence results, the present study shows that lenvatinib treatment is accompanied by increased p53 expression and reduced xCT expression in osteosarcoma cells.

In addition, previous studies have shown that bavachin inhibits STAT3 and upregulates p53, thereby inducing ferroptosis in osteosarcoma cells (47). These findings suggest that suppression of p-STAT3 may activate p53 and inhibit tumor progression. Consistent with this regulatory relationship, the present study showed that lenvatinib decreased p-STAT3 expression and increased p53 expression, whereas Fer-1 reversed these changes. Therefore, we propose that lenvatinib-induced ferroptosis-related changes may be associated with reduced STAT3 phosphorylation, increased p53 expression, and reduced xCT expression in osteosarcoma cells involving the p-STAT3/p53/xCT axis.

Two recent studies have reported that xCT upregulation can inhibit ferroptosis in osteosarcoma cells (4, 20). To further evaluate the involvement of xCT in lenvatinib-induced ferroptosis-related changes, stable xCT-overexpressing osteosarcoma cell models were established. qRT-PCR showed that xCT upregulation significantly decreased p53 and PTGS2 expression and increased GPX4 expression, whereas lenvatinib treatment reversed these effects. Western blotting showed that, after xCT upregulation, total STAT3 expression remained unchanged, p53 expression decreased, and p-STAT3, GPX4, and xCT expression increased. Lenvatinib treatment again reversed these expression patterns. The significant change in the p-STAT3/STAT3 ratio further supports an association between lenvatinib-induced ferroptosis-related changes and modulation of the p-STAT3/p53/xCT axis.

The effects of xCT upregulation on osteosarcoma cell proliferation were also examined. Compared with the oe-NC group, xCT upregulation significantly promoted osteosarcoma cell proliferation, whereas lenvatinib treatment markedly inhibited cell proliferation compared with the untreated group. This finding is consistent with the earlier proliferation assays and indicates that xCT contributes to osteosarcoma cell survival. In addition, because the effect of ferroptosis induction on osteosarcoma cell biological behavior has not been fully clarified, migration and invasion assays were performed in this study. Wound-healing assays showed that xCT upregulation significantly enhanced osteosarcoma cell migration, whereas lenvatinib treatment inhibited this effect. Transwell assays further showed that xCT upregulation significantly promoted both migration and invasion, whereas lenvatinib markedly suppressed these malignant phenotypes. These results indicate that xCT not only protects osteosarcoma cells from ferroptosis but also contributes to migration and invasion, while lenvatinib counteracts these effects.

Several limitations should be acknowledged. First, this study was primarily based on *in vitro* osteosarcoma cell models, and *in vivo* validation using xenograft or orthotopic osteosarcoma models was not performed. Therefore, the translational relevance of lenvatinib-induced ferroptosis-related changes requires further investigation. Second, although Fer-1 rescue, mitochondrial morphological alterations, Fe^2+^ accumulation, GSH depletion, ROS generation, and MDA elevation support the involvement of ferroptosis, additional ferroptosis-specific approaches, such as C11-BODIPY staining, Liproxstatin-1 or DFO rescue, and positive controls such as erastin or RSL3, would further strengthen this conclusion. Third, the current evidence supports an association among p-STAT3, p53, and xCT, but does not fully establish the causal sequence of the p-STAT3/p53/xCT pathway. Future studies using STAT3 activation, p53 knockdown, and rescue assays are needed to verify this regulatory order. Fourth, the clinical pharmacological relevance of the *in vitro* lenvatinib concentrations remains to be further clarified. Finally, the weaker response of MG63 cells compared with 143B and U2OS cells suggests intercellular heterogeneity among osteosarcoma models, which should be considered in future studies. In addition, the p53 status of the osteosarcoma cell lines used in this study was not experimentally verified, and direct transcriptional regulation of xCT by p53 was not assessed.

In summary, this study systematically explored the antitumor effect and molecular mechanism of lenvatinib in osteosarcoma cells. The findings suggest that lenvatinib induces ferroptosis-related changes and suppresses migration and invasion in osteosarcoma cells, at least in part, in association with modulation of the p-STAT3/p53/xCT axis. These findings provide a rationale for further investigation of ferroptosis-related mechanisms and potential therapeutic targets in osteosarcoma. More broadly, these results may offer new insight into improving conventional therapeutic strategies and enhancing the prognosis of patients with osteosarcoma.

## Data Availability

The original contributions presented in the study are included in the article/supplementary material. Further inquiries can be directed to the corresponding authors.
